# Impaired glycosylation promotes rapid transition to hepatocellular carcinoma in model of diet-induced steatotic liver disease

**DOI:** 10.1172/JCI197719

**Published:** 2026-03-10

**Authors:** Abhishek K. Singh, Balkrishna Chaube, Kathryn M. Citrin, Joseph W.M. Fowler, Sungwoon Lee, Jonatas Catarino, James Knight, Sarah C. Lowery, Sonal Shree, Keira E. Mahoney, Nabil E. Boutagy, Inmaculada Ruz-Maldonado, Kathy Harry, Marya Shanabrough, Trenton T. Ross, Stacy A. Malaker, Yajaira Suárez, Carlos Fernández-Hernando, Kariona A. Grabińska, William C. Sessa

**Affiliations:** 1 Department of Pharmacology, Vascular Biology and Therapeutics Program, and; 2 Department of Comparative Medicine, Yale Center for Molecular and Systems Metabolism, Yale University School of Medicine, New Haven, Connecticut, USA.; 3 Department of Biosciences and Bioengineering, Indian Institute of Technology Dharwad, Dharwad, Karnataka, India.; 4 Integrative Cell Signaling and Neurobiology of Metabolism Program, Department of Comparative Medicine, and; 5 Department of Genetics, Yale University School of Medicine, New Haven, Connecticut, USA.; 6 Department of Chemistry,; 7 Department of Molecular Biophysics and Biochemistry, and; 8 Department of Internal Medicine, Yale University, New Haven, Connecticut, USA.; 9 Internal Medicine Research Unit, Pfizer, Cambridge, Massachusetts, USA.; 10 Department of Pathology, Yale University School of Medicine, New Haven, Connecticut, USA.

**Keywords:** Hepatology, Metabolism, Oncology, Lipoproteins, Liver cancer, Mouse models

## Abstract

Obesity-linked steatosis is a significant risk factor for hepatocellular carcinoma (HCC); however, the molecular mechanisms underlying the transition from metabolic dysfunction–associated steatotic liver disease (MASLD) to HCC remain unclear. Here, we explored the role of the ER-associated protein NgBR, an essential component of the *cis*-prenyltransferase (cis-PTase) enzyme, in chronic liver disease. Hepatocyte-specific NgBR deletion in mice (N-LKO) intensified triacylglycerol (TAG) accumulation, inflammatory responses, ER/oxidative stress, and fibrosis, ultimately resulting in HCC development with 100% penetrance after 4 months on a high-fat diet. Similarly, liver-specific knockout of DHDDS, NgBR’s cis-PTase partner, and a knockin model carrying a human NgBR mutation that impairs cis-PTase activity developed HCC under high-fat diet conditions, although with lower penetrance. A single-cell transcriptomic atlas from affected livers provides a detailed molecular analysis of the transition from liver pathophysiology to HCC development. Mechanistically, NgBR deficiency promoted excessive hepatic TAG accumulation by enhancing lipid uptake and impairing VLDL secretion. Importantly, pharmacological inhibition of diacylglycerol acyltransferase-2 (DGAT2), a key enzyme in TAG synthesis, abrogated diet-induced liver damage and HCC burden in N-LKO mice. Overall, our findings establish cis-PTase as a critical suppressor of MASLD-HCC conversion and suggest DGAT2 inhibition may serve as a promising therapeutic approach to delay HCC formation in advanced metabolic dysfunction–associated steatohepatitis.

## Abstract 2

Loss of the liver protein NgBR drives fat buildup, inflammation, and rapid liver cancer in obese mice. Blocking fat synthesis prevents damage, suggesting a promising treatment strategy.

## Graphical abstract

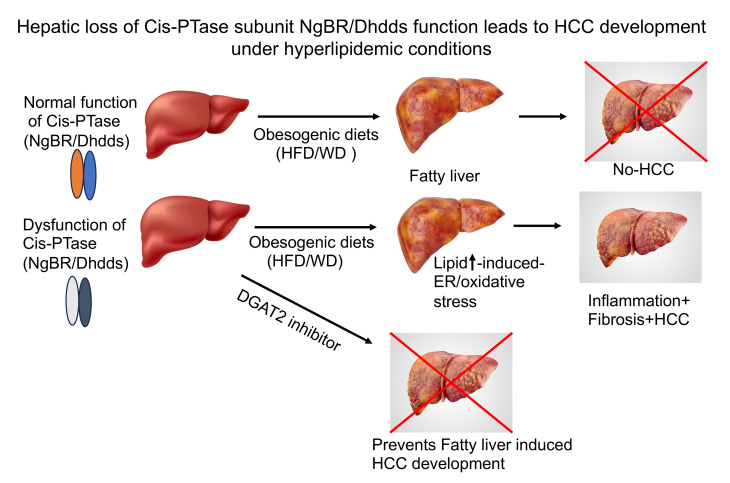

## Introduction

Hepatocellular carcinoma (HCC) typically develops in patients with chronic liver disease resulting from viral (HBV and HCV) or nonviral (alcohol) metabolic dysfunction–associated steatotic liver disease (MASLD) (1, 2). Recent epidemiological studies indicate an increase in HCC incidence in patients with MASLD, particularly in Western countries with elevated prevalence of obesity associated MASLD (3). MASLD is characterized by excessive accumulation of triacylglycerol (TAG) in hepatocytes due to increased fatty acid (FA) uptake, biosynthesis, or imbalanced FA partitioning into storage, oxidative, and lipoprotein secretory pathways (4). Sequential induction of metabolic dysfunction–associated steatohepatitis (MASH) and hepatic fibrosis can occur in MASLD, leading to HCC development.

Despite recent efforts to develop preclinical mouse models for MASH-HCC, including genetic, toxin, or dietary models, these models differ significantly in the cause of liver injury, penetrance, and length of onset of tumor development. Moreover, none of these models fully replicate human MASLD-HCC pathology (5, 6). As a result, effective treatments for patients with MASH-HCC are currently unavailable, primarily due to a lack of precise understanding of the mechanisms underlying obesity-induced HCC in the context of MASLD and the absence of a reliable diet-induced HCC mouse model for hypothesis generation and therapeutic testing.

Previously, we identified the highly conserved, ER-associated heteromeric protein complex responsible for *cis*-prenyltransferase (cis-PTase) activity, consisting of the membrane-associated subunits nuclear undecaprenyl synthase (NUS1), also called NgBR, and dehydrodolichol diphosphate synthase (Dhdds) (7–9). This evolutionarily conserved, heteromeric complex is essential for cis-PTase activity, the rate-limiting enzyme committed to the synthesis of dolichol phosphate, an obligate lipid carrier for protein glycosylation reactions including N-glycosylation, C-mannosylation, O-mannosylation, and glycosylphosphatidylinositol anchor biosynthesis (8, 10, 11). In recent years, the physiological significance of the NgBR/Dhdds complex has received considerable attention due to several in-depth genetic exome sequencing studies reporting various pathogenic mutations in both subunits and evidence that such loss-of-function mutations can cause diseases. Mutations within the genes of either subunit, such as the R290H loss-of-function mutation in NgBR or the K42E mutation in Dhdds, can cause distinct clinical disorders, including severe congenital disorder of glycosylation, retinitis pigmentosa, neurodegenerative diseases, and epileptic encephalopathies (8, 11–13). Global deletion of NgBR in mice leads to early embryonic lethality (E6.5), and conditional deletion in the endothelium causes lethality due to impairment of glycosylation of several critical proteins, resulting in ER stress and impaired cell growth (7, 8). Collectively, these studies show that NgBR is essential for life via the critical role of dolichol-mediated protein glycosylation reactions that regulate the secretion, turnover, and function of many proteins.

Defective or altered protein glycosylation is associated with the development of several cancers, including HCC (14–16), which led us to investigate the role of NgBR in a mouse model of MASLD. Surprisingly, deletion of NgBR in the liver (N-LKO) of mice fed a high-fat diet (HFD), but not a normal chow diet (CD), promoted the hallmarks of steatotic liver disease leading to HCC. This occurred with 100% penetrance after 16 weeks of HFD, and the model exhibited relevant clinical features, including steatotic liver secondary to elevated hepatic lipid uptake and impaired VLDL secretion, T cell recruitment, and fibrosis, ultimately resulting in carcinogenesis. Importantly, pharmacological inhibition of hepatic TAG synthesis in N-LKO mice prevented diet-induced metabolic alterations and HCC development, thus providing support for etiology of fat-driven hepatic carcinogenesis.

## Results

### Liver-specific NgBR deficiency triggers HFD-induced hepatocarcinogenesis.

**Figure 1:**
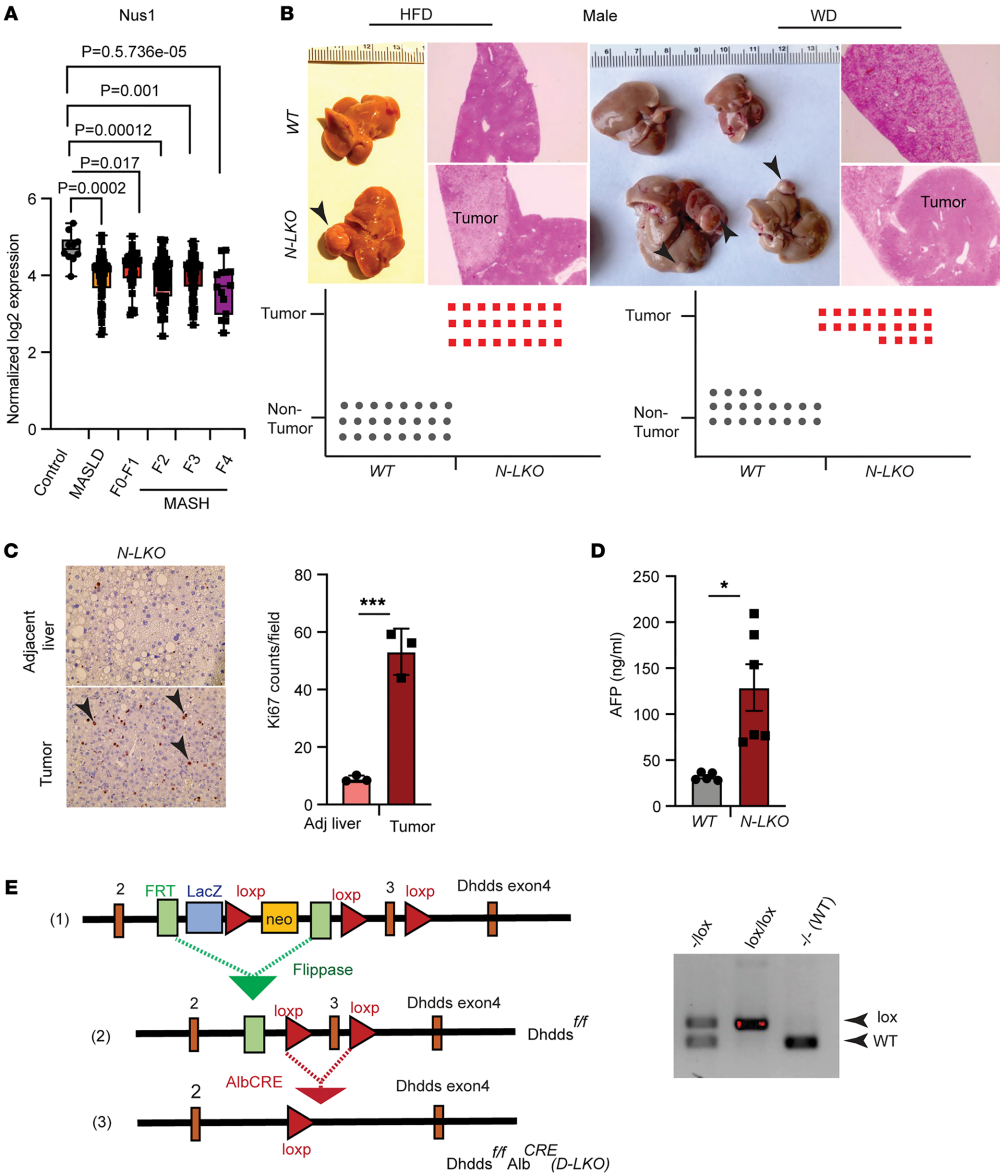
Loss of hepatic NgBR/Dhdds drives HCC development in diet-induced obesity, and reduced NgBR expression correlates with MASLD-MASH progression in human liver. (**A**) Log_2_ normalized mRNA expression levels of NgBR (Nus1) in human liver samples with MASLD and various stages of MASH with different degrees of fibrosis. RNA-seq was performed on 15 healthy liver samples, 30 steatosis samples, and 20 MASH samples (F0–F4). Each data point represents a biological replicate. Statistical comparisons between control and liver disease conditions were conducted using limma’s decideTests with Benjamini-Hochberg correction. The adjusted *P* values are shown on the graph. In the box-and-whisker plots, the central lines represent medians, the edges of the box indicate upper and lower quartiles, and the whiskers indicate the minimum and maximum values. (**B**) Representative liver images from WT and N-LKO male mice fed an HFD or WD for 16 weeks, with arrowheads indicating HCC. Histological analysis of liver and tumor sections stained with H&E is shown on the right, and the graph summarizes HCC incidence in N-LKO and WT mice on HFD (*n* = 24) and WD (*n* = 16) (bottom). Original magnification, ×5. (**C**) IHC analysis of the proliferation marker Ki-67 in tumor and tumor-adjacent liver of N-LKO mice on WD for 16 weeks, with quantification of Ki-67/field (*n* = 3) shown at right. Original magnification, ×20. (**D**) Circulating AFP levels in WT (*n* = 5) and N-LKO (*n* = 6) mice fed HFD. (**E**) Schematic showing the generation of D-LKO mice. (1) The cassette is composed of a short flippase recombination enzyme (Flp) recognition target (FRT) and a Cre recombinase recognition target (loxP). Dhdds exons 2–3 are flanked by the loxP site. (2) Mice with floxed allele but missing neomycin cassette were generated by crossing with Flp recombinase deleter mice. (3) Afterward, these floxed mice were bred with mice expressing Cre recombinase to generate tissue-specific (D-LKO) mice. Genotyping from *Dhdds^fl/fl^* mice presentation bands from 1, both, or none of the floxed alleles. All data are presented as mean ± SEM. Statistical analysis: *P < 0.05 and ***P < 0.001, comparing N-LKO with WT mice using an unpaired 2-sided Welch’s *t* test.

Hepatic abnormalities are frequently observed among patients with congenital disorder of glycosylation, alongside neurological manifestations, muscular dystrophies, and dysmorphism (17–22). These hepatic abnormalities include hepatomegaly, cirrhosis, liver fibrosis, and steatosis. Notably, a prior study identified a significant reduction in liver dolichol levels, the end product of the cis-PTase enzyme subunit NgBR/Dhdds, in individuals with HCC and cirrhosis compared with healthy counterparts (23). As dolichol serves as a critical carrier for protein glycosylation, this finding prompted an inquiry into whether the loss of NgBR in the liver contributes to the development of MASLD-induced HCC. To elucidate this potential link, we initially examined if the transition from a healthy liver to MASLD/MASH affects NgBR levels in humans by analyzing transcriptomic datasets. Furthermore, Western blot analyses were conducted on liver tissue samples obtained from individuals with various steatotic liver diseases, including steatosis, steatohepatitis, and fibrosis. Our findings unveiled a decline in hepatic NgBR transcript levels in patients with MASLD and MASH across different fibrosis stages (F0–F1, F3, and F4) compared with healthy patients (Figure 1A). This decrease was mirrored at the protein level (Supplemental Figure 1, A and B; supplemental material available online with this article; https://doi.org/10.1172/JCI197719DS1), suggesting a potential role for hepatic NgBR inactivation in driving liver cancer, particularly in late-stage MASH (F4), a pre-HCC condition.

To further interrogate this hypothesis, we generated liver-specific NgBR knockout mice (N-LKO) (Supplemental Figure 1, C and D) and subjected them to an HFD or a high-fat, high-cholesterol diet (Western-type diet [WD]) for 16 weeks, which is conducive to obesity-linked steatotic liver. Intriguingly, male N-LKO mice exhibited a 100% incidence of multiple liver tumor nodules upon consumption of either HFD or WD for 16 weeks (Figure 1B). This phenomenon was observed in offspring from at least 3 independent cohorts of mice but was not detected in N-LKO mice fed a regular CD, even when euthanized approximately 20 months after birth (Supplemental Figure 1E), suggesting that hepatic suppression of NgBR leads to the development of liver tumors under obesity-induced steatotic liver conditions but not in lean conditions.

In humans, obesity and insulin resistance are commonly associated with MASLD (24, 25). Studies have demonstrated that HFD/WD can induce obesity and insulin resistance in mice as well. In our study, N-LKO mice fed a WD for 16 weeks showed significant weight gain and elevated fasting blood glucose levels compared with those fed a CD, indicating obesity and insulin resistance (Supplemental Figure 1, F–H).

After measuring hepatic tumor nodules (Supplemental Figure 1I), histological analysis revealed that liver tumors consisted of both the fatty transformation of cells as well as solid growth of tumors with distinct margins (Figure 1B). Next, we examined hepatic cell proliferation in tumors by Ki-67 staining. Compared with the adjacent nontumorous liver, the tumor cells showed markedly increased levels of Ki-67 (Figure 1C), resembling MASLD-initiated HCC. Consistent with the development of HCC, alpha fetoprotein (AFP), a plasma biomarker for proliferating hepatocytes and HCC, was significantly elevated in HFD-fed N-LKO mice (Figure 1D). Assessment of blood cell counts after HFD for 16 weeks revealed that circulating WBCs/lymphocytes were significantly increased in N-LKO mice (Supplemental Figure 1J), and the proportion of other circulating cells was similar in both groups of mice (Supplemental Figure 11J). Profiling of lymphocyte subsets showed that CD8^+^ T cells were increased amongst the lymphoid population in N-LKO mice fed an HFD (Supplemental Figure 1K).

To independently validate the concept that a loss of cis-PTase activity functions as a driver of advanced liver disease, we took 2 additional approaches to reduce cis-PTase activity in mice. Previously, it was shown that a NgBR R290H mutation in humans is a loss-of-function mutation by virtue of this amino acid substitution impairing substrate binding and enzymatic function of the NgBR/DHDDS complex, causing a severe congenital disorder of glycosylation (8). Thus, we generated NgBR orthologous R294H mutant knockin mice (Supplemental Figure 2A). Homozygous R294H NgBR mice died approximately 4–12 weeks after birth, while heterozygous R294H NgBR mice were born with normal frequencies (Supplemental Figure 2B). Interestingly, HCC developed in heterozygous NgBR R294H mutant mice (incidence of ~20%) fed an HFD for 16 weeks (Supplemental Figure 2, C–E). Next, we generated mice lacking hepatic Dhdds (D-LKO) (Figure 1E and Supplemental Figure 2F), an obligate subunit of cis-PTase enzyme that heterodimerizes with NgBR. Feeding D-LKO mice an HFD for 16 weeks also promoted HCC with a frequency of approximately 40% (Supplemental Figure 2G), suggesting a critical role of cis-PTase activity in preventing hepatocyte transformation when exposed to a lipid-rich diet. Collectively, our data demonstrate that hepatic loss of function of the heteromeric NgBR/DHDDS cis-PTase complex in 3 distinct genetic models directly contributes to liver carcinogenesis after a short duration of feeding mice an HFD.

### Hepatic NgBR deficiency upregulates gene expression profiles associated with HCC and promotes TAG accumulation, inflammation, and fibrosis in the liver.

To assess global pattens of gene expression, we performed unbiased RNA-seq analysis on RNA isolated from livers of N-LKO and WT mice fed an HFD/WD for 16 weeks. Multidimensional scaling plot analysis of HFD-fed mice revealed a clear separation of gene expression patterns based on genotype (Supplemental Figure 3A). In N-LKO mice, 697 genes were differentially regulated, with 407 upregulated and 290 downregulated (Supplemental Figure 3A). KEGG pathway analysis identified numerous genes involved in steatotic liver formation, ROS production, DNA damage, and cancer development that were upregulated in N-LKO mice (Supplemental Figure 3B). Several genes of these pathways are thought to play a critical role in HCC development and may contribute to impaired liver functions and tumor formation in N-LKO mice. Heatmap analysis of tumors from WD-fed N-LKO mice confirmed upregulation of *Afp* and *Fabp5* and downregulation of *Pcsk9*, markers associated with tumor development (Supplemental Figure 3C).

**Figure 2:**
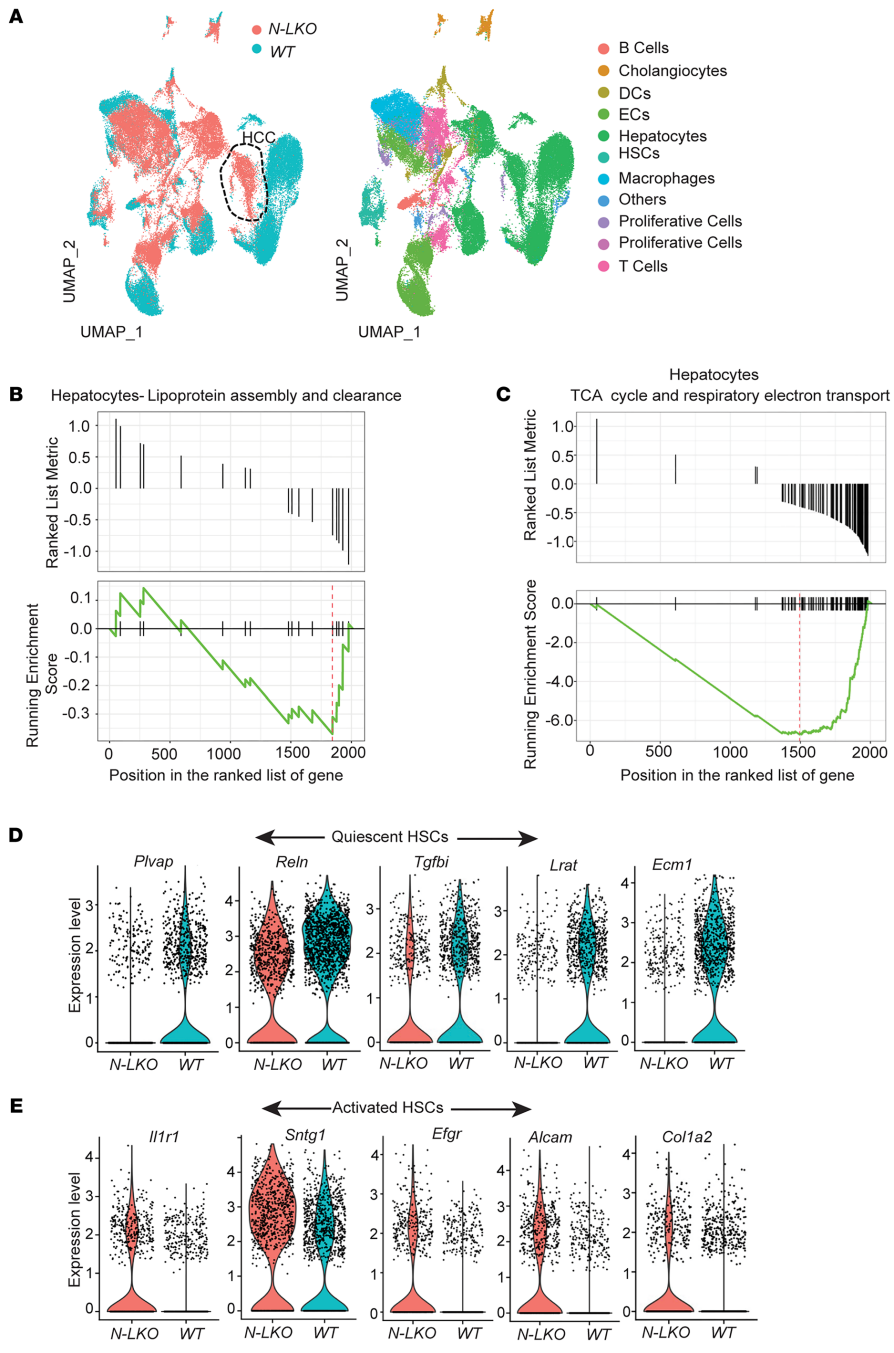
Liver-specific NgBR depletion induces gene expression patterns associated with MASLD and HCC in hepatic cells. (**A**) scRNA-seq of the livers isolated from WT and N-LKO mice using the 10x Genomics Chromium platform. UMAP visualized clustering of liver single-cell transcriptomes (7,000 cells from WT and 7,000 cells from N-LKO mice fed a WD). Colors represent cell types or genotypes; the circle indicates cancer cells. ECs, endothelial cells. (**B** and **C**) Enrichment score curves and GSEA plots of significant differentially expressed genes between WT and N-LKO hepatocytes. The peak in the plot shows the downregulation of the gene sets associated with lipoprotein packaging and secretion and mitochondrial oxidative function. (**D** and **E**) Violin plots comparing the expression levels of HSC quiescent marker genes (top) and activation marker genes (bottom) that are downregulated and upregulated, respectively, in N-LKO mice relative to WT mice. The plots illustrate a significant shift in the phenotype of HSCs in N-LKO mice, as demonstrated by the altered expression of quiescent and activation markers.

To investigate liver cell heterogeneity and dynamic alterations during MASLD-HCC pathogenesis, scRNA-seq on parenchymal cells (PCs) and nonparenchymal cells (NPCs) or tumor cells isolated from the livers of WT or N-LKO mice fed a WD for 16 weeks was performed. Data analysis identified 33 discrete cell populations clustered into 11 unique cell types, including hepatocytes, hepatic stellate cells (HSCs), cholangiocytes, endothelial cells, macrophages, DCs, Kupffer cells, T cells, and B cells (Figure 2A and Supplemental Figure 4A). By delineating component cell analysis, a distinctive transcriptional signature of PCs and NPCs in N-LKO compared with WT mice identified 9 distinct hepatocyte clusters, comprising Hep1–Hep9, with 2 unique hepatocyte clusters observed only in N-LKO mice (Supplemental Figure 4, A–C). Furthermore, the Hep4 population in N-LKO mice was enriched for HCC, as confirmed by high expression of cytokeratin genes such as Krt8, Krt18, Spp1, and Epcam (Figure 2A). GSEA revealed that the hepatocytes in N-LKO mice had decreased lipoprotein metabolism, mitochondria function, and oxidative stress response compared with WT mice (Figure 2, B and C, Supplemental Figure 4D, and Supplemental Figure 5, A–D). Additionally, there was marked upregulation of genes associated with ER stress (Supplemental Figure 5E), consistent with loss of NgBR, as previously described (7). We also identified a decrease in growth suppressors and cell cycle checkpoint enriched genes, such as *Apc*, *p53*, and *Pten* (Supplemental Figure 6, A–F), and enhanced expression of genes related to oncogenic signaling pathways, including receptor tyrosine kinase signaling (*Egfr*, *Fgfr2*, *Insr*, *Nrp*, and *Braf*), Rac/Rho GTPases, VEGF signaling, and extracellular matrix remodeling (Supplemental Figure 7, A–H). These findings collectively suggest that the observed changes in gene expression may contribute to the development of cancer in N-LKO mice.

We also investigated other important NPCs in the liver, particularly T cells, liver sinusoidal endothelial cells (LSECs), and HSCs, in both WT and N-LKO mice. Analysis showed that there is a marked difference in gene expression among all 4 T cell subclusters in N-LKO mice compared with WT mice (Supplemental Figure 8, A–C). In addition, a notable increase in T cell markers across all clusters exhibiting reduced enrichment of genes linked to FA metabolism, mitochondrial function, and oxidative stress response was found in N-LKO mice (Supplemental Figure 8, D and E). This rise was accompanied by an increase in T cells expressing markers of stress and exhaustion (Supplemental Figure 8, F and G), suggesting chronic inflammation, a hallmark of MASH. Analysis of LSECs revealed a distinct separation of gene expression profiles across 3 LSEC subclusters (Supplemental Figure 9, A–C). GSEA indicated that gene expression involved in essential cellular oxidative processes including the citric acid (TCA) cycle, FA oxidation, respiratory electron transport, ATP synthesis, and cellular stress responses was markedly downregulated in N-LKO mice (Supplemental Figure 9, D–F). In contrast, gene expression related to extracellular remodeling and inflammation was significantly upregulated (Supplemental Figure 9, G–J), suggesting impaired LSEC function and a shift toward pathological features associated with chronic steatotic liver disease. Furthermore, analysis of HSCs identified a subcluster (Supplemental Figure 10, A and B) that had reduced expression of HSC quiescent markers, lipid metabolism–related genes, and oxidative stress response genes in N-LKO mice compared with the control group (Figure 2, D and E, and Supplemental Figure 10, C–E). In contrast, this subcluster of HSCs exhibited higher expression of markers associated with HSC activation and genes enriched in extracellular matrix remodeling (Figure 2E and Supplemental Figure 10, F–H), indicating that the N-LKO mice may have been at a higher risk for liver fibrosis. Collectively, these changes in impaired liver lipoprotein metabolism, oxidative/ER stress, inflammation, and fibrosis may contribute to liver dysfunction and HCC formation observed in these mice.

### Hepatic NgBR suppression promotes TAG accumulation, inflammation, and fibrosis in the liver.

**Figure 3:**
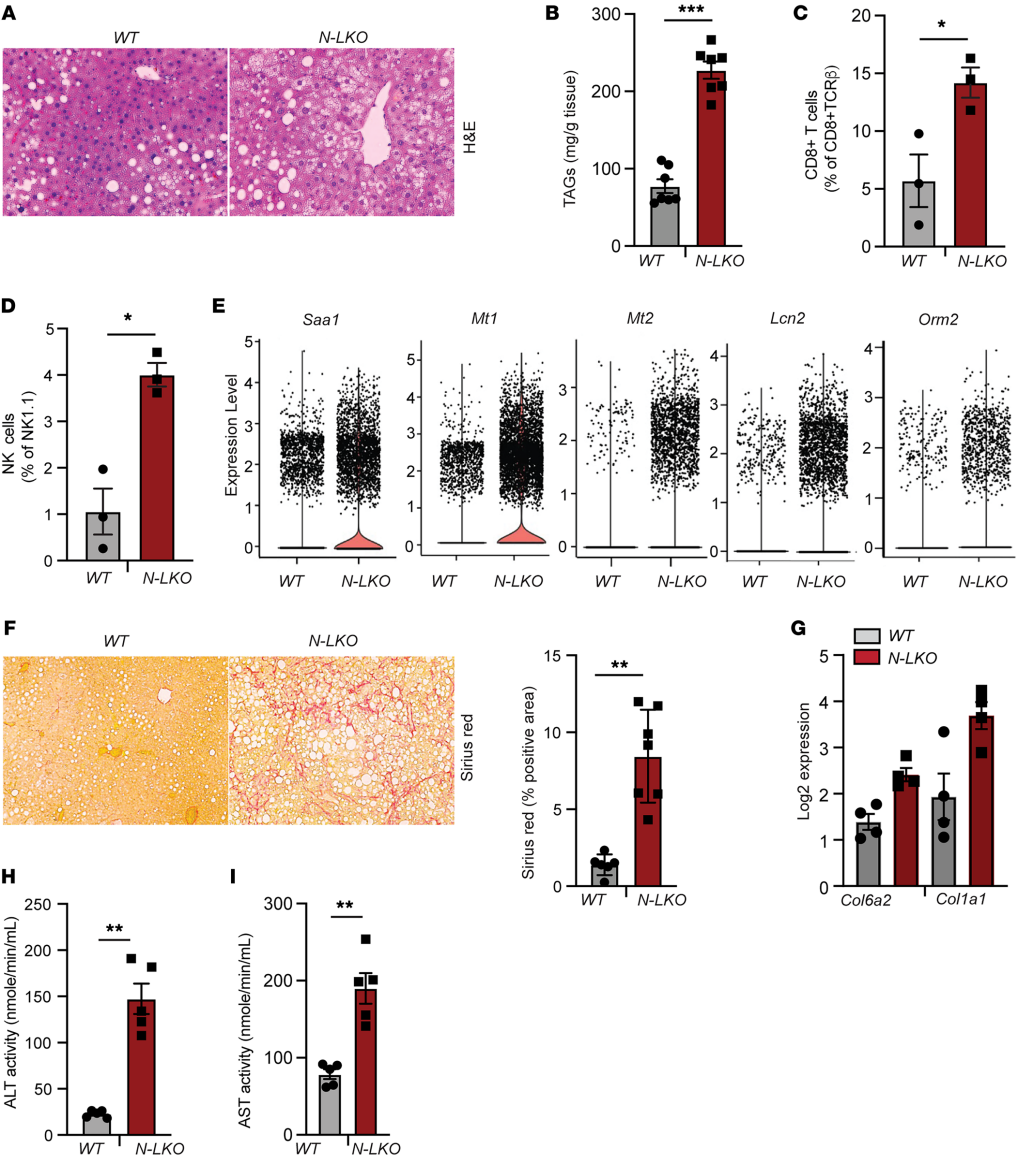
Hepatic NgBR deficiency exacerbates MASH fibrosis phenotype in response to overnutrition. (**A** and **B**) Representative images of liver sections stained with H&E (**A**) and hepatic TAG levels (**B**) in WT and N-LKO mice fed an HFD for 16 weeks (*n* = 7). Original magnification, ×40. (**C** and **D**) Liver lymphoid cells, including CD8^+^ T cells and NK cells (*n* = 3), isolated from WT and N-LKO mice fed an HFD for 16 weeks assessed by flow cytometry. (**E**) Violin plots illustrating that the expression of acute phage response genes is elevated in the hepatocytes of N-LKO mice compared with WT mice fed a WD. (**F**) Representative images and quantification of Sirius red staining in the liver sections from WT and N-LKO mice fed an HFD for 16 weeks (*n* = 7). Original magnification, ×20. (**G**) Log-transformed mRNA expression of Col6a2 and Col1a1 from liver of WT and N-LKO mice fed an HFD for 16 weeks (*n* = 4). (**H** and **I**) Plasma ALT and AST levels in WT and N-LKO mice fed an HFD (*n* = 5). All data are presented as mean ± SEM. Statistical analysis: *P < 0.05, **P < 0.01, and ***P < 0.001, comparing N-LKO with WT mice using an unpaired 2-sided Welch’s *t* test.

Utilizing scRNA-seq, we identified that NgBR deficiency in mice liver led to impaired lipid metabolism pathways, augmented immune cell infiltration markers, and upregulated expression of genes related to ER and oxidative stress as well as fibrosis. These are important characteristics of MASLD-derived HCC (26). To further explore the functional consequences of these observations, we characterized the steatotic liver phenotype and specific immune cell subsets present in the liver of N-LKO mice subjected to a 16-week HFD regimen. Consistent with previous reports (27, 28), N-LKO mice fed an HFD displayed extensive hepatic TAG accumulation (Figure 3, A and B). HFD-fed N-LKO mice exhibit significantly increased hepatic infiltration of lymphocytes such as CD8^+^ T and NK cells, and myelocytes, including macrophages, DCs, and neutrophils, while B cells and monocytes showed no alteration and CD4^+^ T cells displayed an upward trend (Figure 3, C and D, and Supplemental Figure 11, A–F). Previous studies have reported that inflammation found in steatotic liver disease is accompanied by the hepatic infiltration of resident CD8^+^ T cells that express programmed death protein (PD-1) (29), thus suppressing the anticancer action of cytotoxic CD8^+^ T cells and driving the development of HCC. Indeed, CD8^+^ T cells that express elevated levels of PD-1 are found in the liver of HFD-fed N-LKO mice (Supplemental Figure 11G). Furthermore, scRNA-seq analysis in hepatocytes demonstrated a significant elevation in acute phase response genes, including Saa1, Mt1, Mt2, Lcn2, and Orm2, which arise from inflammation in N-LKO mice compared with WT mice fed a WD (Figure 3E), consistent with previous reports (30, 31). Concomitantly, N-LKO mice fed an HFD for 16 weeks had increased liver damage, as shown by increased hepatic collagen deposition and fibrosis (Sirius red staining), augmented expression of fibrotic genes, and elevated levels of the liver-damaging enzymes alanine aminotransferase (ALT) and aspartate aminotransferase (AST) in the plasma, markers of liver damage resembling some key patterns in human MASH (Figure 3, F–I). Excessive hepatic lipid accumulation increases inflammation, fibrosis, and oxidative DNA damage caused by elevated ROS production, lipid peroxidation, and ER stress, well-established drivers of liver damage (32–34). N-LKO mice fed an HFD showed significantly increased hepatic ROS production, malondialdehyde (MDA) content (final oxidative product of polyunsaturated FA peroxidation), and key ER stress markers such as ATF4 (Supplemental Figure 12, A–C). Collectively, these results imply that hepatic suppression of NgBR contributes to the aggravation of MASLD, MASH, and fibrosis, which may account for the HCC development.

### Loss of NgBR function in the liver enhances hepatic lipid uptake and suppresses VLDL production, leading to steatotic liver development.

We observed reduced NgBR expression in the liver of patients with MAFDL/MASH compared with healthy ones and HCC development in mice with liver-specific loss of NgBR under conditions of steatotic liver induced by an HFD/WD but not under normal CD conditions, suggesting a potential link between steatotic liver and HCC development in the absence of functional hepatic NgBR. Therefore, we hypothesize that steatotic liver may contribute to HCC onset when hepatic NgBR is not functioning properly. To investigate this hypothesis, our first goal was to explore the function of NgBR. We sought to determine whether defects in dolichol biosynthesis and protein glycosylation were linked to NgBR function loss, as previously observed in cases of global NgBR deficiency (8) and in endothelial cell–specific knockout mice (7). To achieve this, we assessed cis-PTase enzymatic activity and global surface protein glycosylation in hepatocytes using flow cytometry with wheat germ agglutinin (WGA) lectin affinity staining. As expected, hepatocytes from N-LKO mice showed significantly reduced cis-PTase activity, confirming enzymatic loss (Supplemental Figure 13A). WGA binding was also reduced by approximately 30% in both N-LKO hepatocytes and NgBR-depleted human Huh7 cells (Supplemental Figure 13, B–D), indicating impaired glycosylation due to hepatic NgBR loss.

Despite this reduction, the moderate (~30%) glycosylation deficit may be partially compensated for by NgBR-expressing liver-resident cells (e.g., endothelial, stellate, and Kupffer cells) that could supply dolichol to hepatocytes. Additionally, dolichol transport via HDL particles abundant in the blood/serum and for which the liver is a major sink (35) may further mitigate glycosylation loss.

To explore this compensatory mechanism, we cultured N-LKO hepatocytes in serum-free media. These cells failed to survive without serum but remained viable when serum was present, likely due to HDL-derived dolichol. Notably, dolichol-P supplementation rescued cell survival even in serum-free conditions, reinforcing its role in glycosylation and hepatocyte viability (Supplemental Figure 13E).

Overall, these findings highlight the essential role of NgBR in dolichol-mediated glycosylation and suggest that compensatory dolichol sources from other hepatic cells and circulation support hepatocyte survival in liver-specific NgBR-knockout mice. In contrast, such compensation is likely absent in global or endothelial-specific knockouts, potentially explaining their lethality. Thus, hepatocyte-specific NgBR depletion leads to reduced cis-PTase activity and impaired global protein glycosylation, indicating a loss-of-function effect of NgBR in hepatocytes.

**Figure 4:**
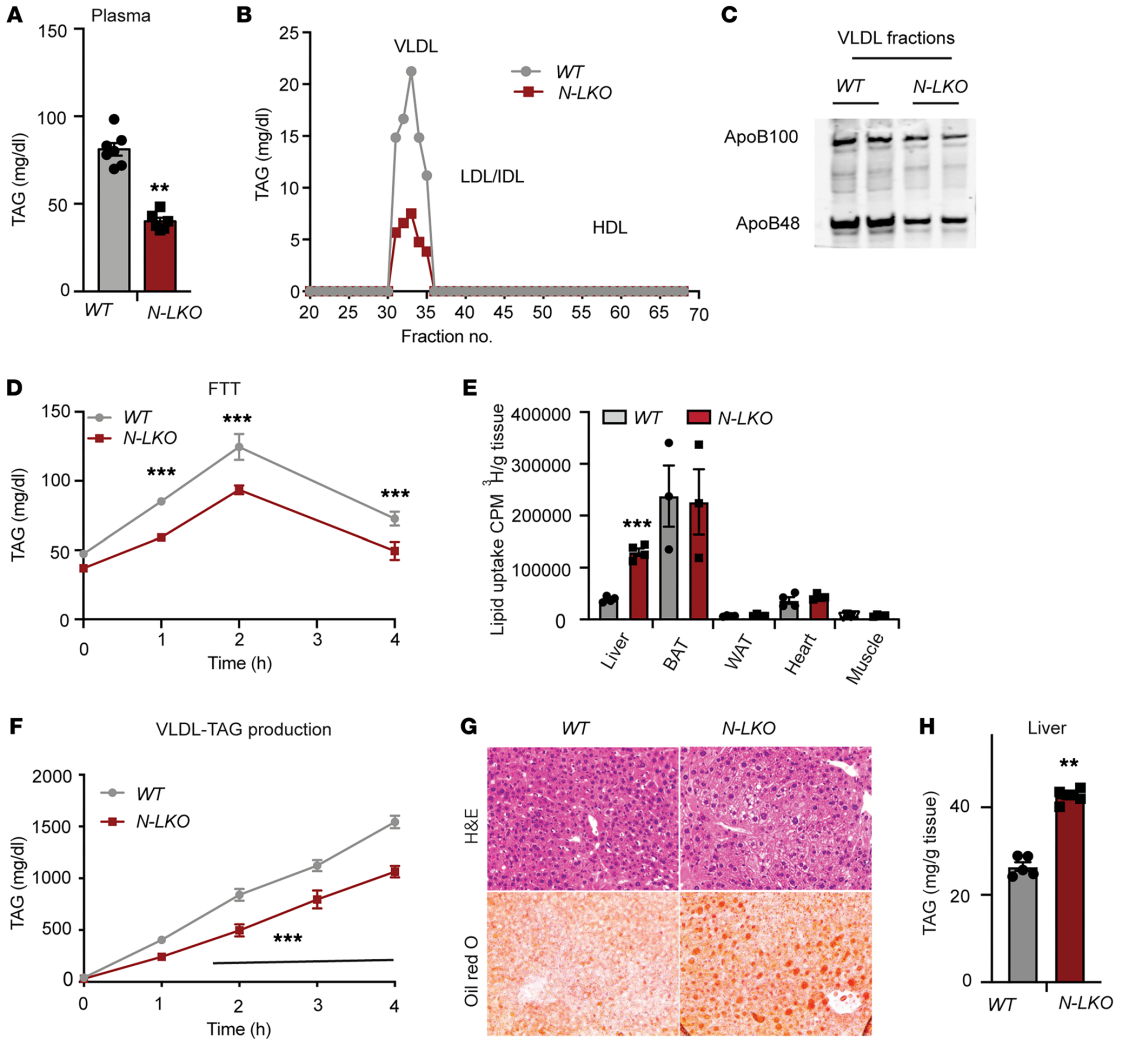
Loss of NgBR function in the liver enhances lipid uptake and impairs VLDL-TAG secretion. (**A**) Circulating TAG levels in overnight-fasted WT and N-LKO mice on a 6-month CD (*n* = 6). (**B**) TAG content of FPLC-fractionated lipoproteins from pooled plasma of overnight-fasted WT and N-LKO mice fed a CD for 6 months (*n* = 5). (**C**) Western blot analyses of plasma ApoB100 and ApoB48 in the VLDL fractions from FPLC-fractionated lipoproteins (*n* = 5). (**D**) Oral lipid tolerance test: TAG clearance from the plasma of WT and N-LKO mice, fasted for 4 hours, then given an oral gavage of olive oil (*n* = 5). (**E**) Radiolabeled triolein uptake in various tissues 2 hours after oral gavage in WT and N-LKO mice fasted for 6 hours (*n* = 3–4). BAT, brown adipose tissue; WAT, white adipose tissue. (**F**) Plasma TAG levels in overnight-fasted WT and N-LKO mice treated with lipoprotein lipase inhibitor 407 to block lipolysis of circulating TAG-rich lipoprotein (*n* = 5). (**G**) Representative images of H&E-stained and Oil Red O–stained liver sections. Original magnification, x40. (**H**) Hepatic TAG levels in WT and N-LKO mice fed a CD for 6 months (*n* = 4). Statistical analysis: **P < 0.01 and ***P < 0.001 using a 2-sided Welch’s *t* test (**A**, **E**, and **H**) and ***P < 0.001 using 2-way ANOVA with Šidák’s multiple-comparison test (**F**).

To understand how hepatic NgBR dysfunction contributes to steatosis, we examined circulating TAG levels in N-LKO mice fed either a standard CD or an HFD. Notably, we observed significantly reduced plasma TAG levels in fasting N-LKO mice, regardless of diet type (Figure 4A and Supplemental Figure 14A). Fractionation analysis of plasma using fast protein liquid chromatography (FPLC) revealed diminished TAG levels specifically in VLDL fractions (Figure 4B and Supplemental Figure 14B). Correspondingly, levels of apolipoproteins ApoB100 and ApoB48, assessed in isolated VLDL fractions, were lower in N-LKO compared with WT mice (Figure 4C). To discern whether the decreased plasma TAG levels in N-LKO mice stemmed from heightened tissue lipid uptake or impaired hepatic VLDL secretion, we conducted several functional assays. These included fat tolerance tests and radiolabeled triolein experiments to evaluate lipid uptake, as well as experiments inhibiting peripheral lipoprotein lipase activity with Poloxamer to probe VLDL-TAG export. Remarkably, all assays consistently indicated increased lipid uptake within the liver and a simultaneous reduction in VLDL-TAG secretion (Figure 4, D–F). In parallel, analysis of gene expression patterns in N-LKO mice fed different diets provided further insights. Notably, N-LKO mice fed a CD exhibited upregulation of Cd36 mRNA, a FA transporter, correlating with enhanced lipid (FA) uptake in the liver (Supplemental Figure 15A). Conversely, N-LKO mice on an HFD showed a decrease in ApoB transcript levels, a gene associated with lipoprotein transport (Supplemental Figure 15B). scRNA-seq data supported these findings, demonstrating reduced expression of genes involved in lipoprotein assembly and VLDL protein secretion in hepatocytes of N-LKO mice on a WD (Figure 2B). These observations align with the diminished VLDL-TAG export observed in functional assays. Further reinforcing these findings, in vitro experiments using Huh7 cells with NgBR knockdown consistently showed heightened FA uptake and lipid accumulation (Supplemental Figure 14, C and E). To ascertain the impact of elevated hepatic lipid uptake and decreased VLDL-TAG secretion on liver steatosis under physiological conditions, we analyzed liver triglyceride levels in N-LKO mice fed a CD for 24 weeks. Indeed, N-LKO mouse livers exhibited visible mild steatosis, as confirmed by an approximately 2-fold increase in hepatic TAG content compared with that in WT mice (Figure 4, G and H).

Considering previous studies proposing LXR-mediated de novo lipogenesis (DNL) as a mechanism for hepatic steatosis in NgBR deficiency (27), we examined the expression levels of key LXR target genes under distinct dietary conditions. However, our results showed consistent expression profiles for these genes in both WT and N-LKO mice, irrespective of their diets, diverging from previous reports (Supplemental Figure 15, C and D).

Given that N-LKO mice fed an HFD/WD exhibited hepatic ER stress and considering prior findings that suppression of NgBR induces ER stress (7), we investigated whether similar stress responses occur under basal conditions. To this end, we examined hepatic expression of ER stress–related genes at the mRNA level using RNA-seq in WT and N-LKO mice fed a CD. Unlike the HFD/WD-fed N-LKO mice, we did not observe any significant differences in ER stress gene expression between WT and N-LKO mice under chow-fed conditions (Supplemental Figure 15E). These findings suggest that hepatic NgBR suppression induces ER stress specifically under lipid overload conditions, but not under basal dietary conditions.

In conclusion, our comprehensive analysis combining in vitro and in vivo experiments suggests that the loss of hepatic NgBR function disrupts lipid homeostasis. This disruption leads to heightened lipid uptake and decreased lipid export from the liver, resulting in TAG accumulation, independent of LXR-mediated DNL or basal ER stress. Such extensive TAG accumulation in the liver may potentially drive the development of HCC in the absence of NgBR function.

### DGAT2 inhibition blocks lipid-induced HCC development in N-LKO mice.

**Figure 5:**
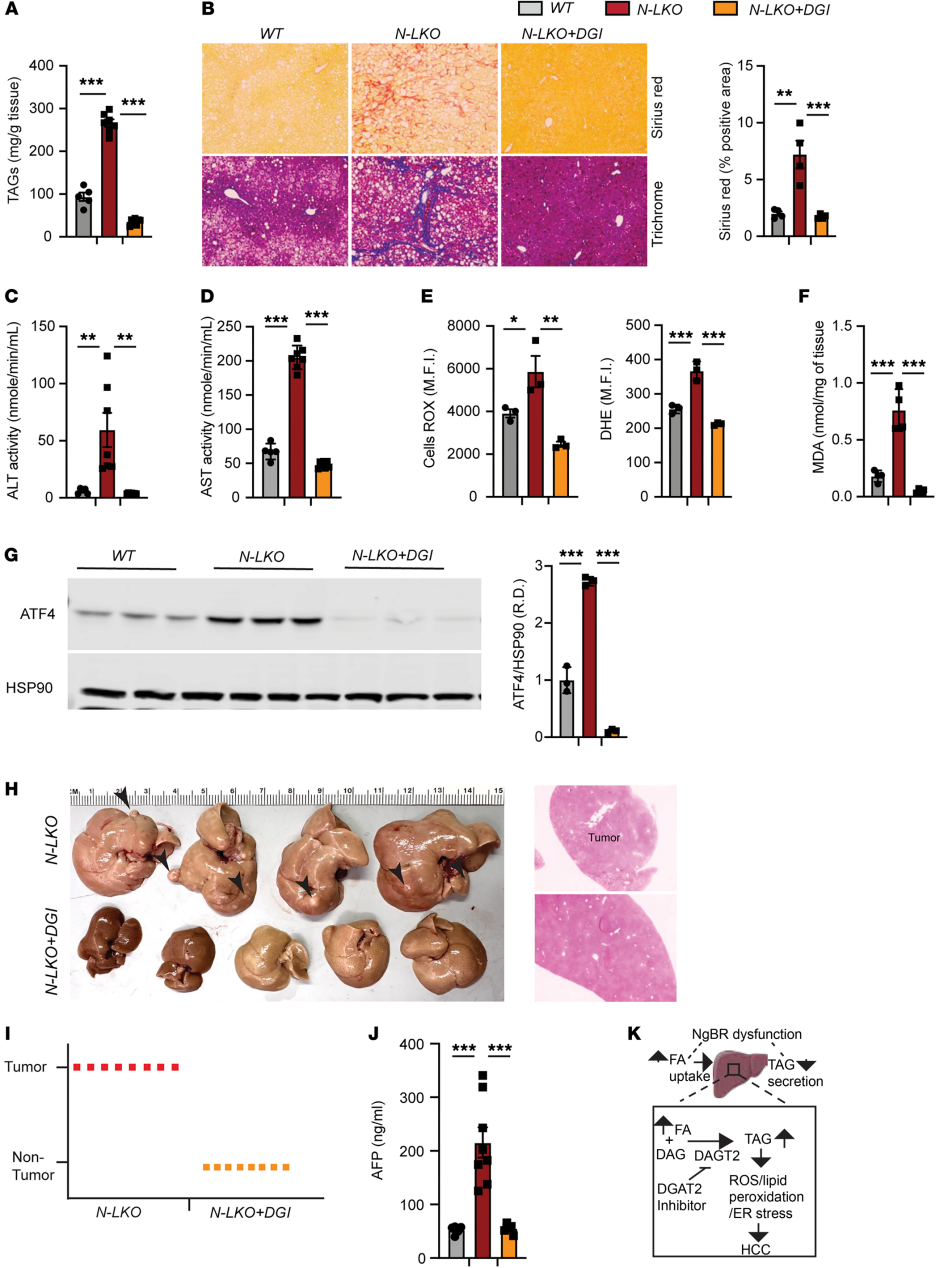
DGAT2 inhibitor treatment prevents diet-induced MASLD-HCC pathogenesis. This study involved 3 groups of mice: WT mice on a WD for 16 weeks, and N-LKO mice divided into 2 subgroups — 1 on WD only and the other on WD with a DGAT2 inhibitor (N-LKO+DGI) for 16 weeks. After 16 weeks, the following parameters were measured. (**A**) Hepatic TAG levels (*n* = 5). (**B**) Representative images and quantification of Sirius red and trichrome staining in liver sections (*n* = 5). Original magnification, x20. (**C** and **D**) Serum ALT and AST levels (WT, *n* = 5; N-LKO, *n* = 6; N-LKO+DGI, *n* = 7). (**E**) Analysis of cellular ROS in primary hepatocytes (*n* = 3). (**F**) Membrane lipid peroxidation via MDA assay in the liver (*n* = 4). (**G**) Western blot and densitometric analysis of ER stress proteins ATF4 and HSP90 (*n* = 3). (**H** and **I**) Photographs of livers from N-LKO and N-LKO+DGI mice, with H&E-stained liver and tumor sections, and a graph summarizing tumor occurrence (*n* = 8). Original magnification, x5. (**J**) Plasma AFP levels (WT, *n* = 5; N-LKO, *n* = 8; N-LKO+DGI, *n* = 8). Data are presented as mean ± SEM, with each point representing a biological sample. Statistical analysis: 1-way ANOVA with Tukey’s test (*P < 0.05, **P < 0.01, and ***P < 0.001). (**K**) Proposed mechanism illustrating the role of liver-derived NgBR in hepatic lipid metabolism.

Hepatic TAG levels are regulated by 4 primary metabolic pathways (36). The main contributor, accounting for about 70% of hepatic TAG, is the uptake of plasma FAs from adipose tissue (37). The second pathway, DNL, synthesizes TAG from acetyl–coenzyme A, mainly from glucose metabolism, contributing 5%–30% of hepatic TAG (37). The other 2 pathways are FA oxidation and TAG secretion within VLDL. Interestingly, in the context of hepatic NgBR depletion, LXR-mediated hepatic DNL was not observed. However, in the absence of hepatic NgBR, we noted an increase in lipid (FA) uptake accompanied by a simultaneous reduction in VLDL TAG export. This suggests that heightened FA uptake, contributing to hepatic TAG synthesis, and decreased VLDL TAG export may be potential mechanisms underlying extensive hepatic TAG accumulation in N-LKO mice. Such accumulation may induce cellular reprogramming favoring DNA damage and HCC development under conditions of lipid overload induced by an HFD/WD. To directly examine if attenuation of liver TAG accumulation impacts the progression to HCC, N-LKO mice were treated with a small molecule inhibitor (PF-06424439) of diacylglycerol acyltransferase-2 (DGAT2). In rodents and primates, DGAT2 catalyzes the final rate-limiting reaction of TAG biosynthesis by incorporating the last fatty acyl group to diacylglycerides (38). In preclinical studies, Pfizer’s PF-06424439 decreased circulating TAG levels and reduced hepatic fat in rodents with MASLD (39). Thus, 8-week-old N-LKO mice were fed a WD formulated with or without PF-06424439 for 16 weeks. Administration of PF-06424439 in N-LKO mice (N-LKO+DGI) markedly reduced hepatic TAG contents and fibrosis (Figure 5, A and B), consistent with previous reports in rodents and humans (38, 39). PF-06424439–treated N-LKO mice were also protected against WD-induced liver damage, as shown by lower circulating levels of liver enzyme ALT and AST (Figure 5, C and D). We next determined whether lowering TAG levels in the liver by DGAT2 inhibition attenuates lipid-mediated deleterious effects in NLKO mice fed a WD. PF-06424439 treatment in NLKO mice fed a WD significantly reduced hepatic ROS production, MDA content, and ER stress marker such as ATF4 (Figure 5, E–G) and completely abolished HCC development and circulating AFP levels (Figure 5, H–J).

Collectively, these results show that the loss of NgBR and a reduction in cis-PTase activity in the liver promote elevated hepatic TAG levels and many of the hallmarks of MASLD driving liver cancer in a rapid, highly penetrant manner. Pharmacological inhibition of DGAT2 restores normal physiological liver lipid homeostasis, resulting in comprehensive prevention of HCC development in NLKO mice. These findings support and give credence to the possibility that reducing liver fat accumulation may impact the transition from MASLD to HCC.

## Discussion

The development of HCC in patients with steatotic liver, especially in late-stage MASH, is a major concern. The mechanisms behind this progression are not fully understood and are difficult to model experimentally. Our study found that liver-specific deletion of NgBR aggravated HFD-driven inflammation, oxidative and ER stress, and fibrosis, leading to liver cancer with 100% incidence, providing a robust model of MASLD-HCC transition. These phenotypes did not occur in NgBR-deficient mice on a normal CD, and HFD-driven HCC was attenuated by pharmacologic inhibition of DGAT2, suggesting that cis-PTase activity is crucial in suppressing MASLD-linked HCC by maintaining liver TAG homeostasis.

Mechanistically, since hepatic NgBR deficiency reduces glycosylation of many intracellular and secreted proteins leading to the unfolded protein response, a synergy of multiple hits triggered by HFD may explain the highly penetrant phenotype. In the case of lipid overload, an increased uptake of lipids along with a decrease in the secretion of VLDL can lead to abnormal lipid accumulation in the liver, triggering the production of ROS and lipid peroxidation, causing oxidative stress. These processes are substantiated using unbiased approaches like scRNA-seq, bulk RNA-seq, and functional assays. Furthermore, suppressing NgBR can lead to reduced protein glycosylation in the ER, which impairs protein folding and triggers ER stress. Lipid-induced oxidative stress exacerbates immune cell infiltration and exhaustion, promoting inflammation and liver fibrosis, as evidenced by unbiased scRNA/bulk RNA-seq and functional assays. The combined effects of increased oxidative and ER stress can lead to DNA damage and mutations, ultimately resulting in the development of liver cancer under conditions of hepatic inflammation and fibrosis.

Indeed, the hepatic loss of NgBR leads to increased liver TAG accumulation as the primary cause of liver pathology and HCC, since blocking TAG synthesis with a DGAT2 inhibitor prevented HCC development and largely dampened the severity of lipotoxic-induced liver damage and fibrosis, demonstrating that TAG accumulation is the primary driver of HCC development in this model (Figure 5K). Hence, this study offers a compelling basis for advancing the development of DGAT2 inhibitors as potential therapeutic agents for addressing the transition from MASLD/MASH to HCC.

In recent years, various diet-induced HCC mouse models have been reported, including choline-deficient HFD (CDHFD) (40), high fat/sugar diets that induce spontaneous MASH-HCC in male B6/129 mice (41), chemical-induced models (42), and hepatocyte-specific deletion or overexpression models of PTEN (43, 44) or urokinase plasminogen activator (45). However, these models have limitations, such as low penetrance of liver tumor incidence, liver toxins, prolonged duration, and mixed genetic backgrounds. Notably, the CDHFD model, while serving as the primary model for TAG-dependent HCC induction, exhibits a limitation with only a 20% HCC incidence over approximately 12 months (40). In addition, some models, such as DEN and CCl4, cause liver damage in a uniform manner (46, 47) that may not replicate the complex and dynamic progression of MASLD-MASH.

To address these limitations, our study provides a reliable and efficient diet-induced robust HCC mouse model with 100% penetrance in a short duration of 4 months, overcoming the limitations of previous models. This model offers potential targets for therapeutic intervention and provides a valuable tool for gaining insights into the mechanisms underlying HCC development. Additionally, using a pure C57BL/6J background in our model allows for more accurate crossbreeding with other transgenic mice and comparisons with other obesity models.

Overall, our findings provide significant insights into the pathogenesis of MASLD-MASH-HCC and suggest potential therapeutic targets for this disease. The reliable diet-induced HCC mouse model presented in this study could facilitate better understanding of obesity-induced HCC prevention and provide a valuable tool for advancing research in this field. Our study holds promise for developing effective strategies for preventing and treating this potentially lethal disease.

This study has enhanced our understanding of how cis-PTase activity and protein glycosylation impact metabolic balance, but several important questions remain. NgBR/Dhdds plays a key role in protein N- and O-linked glycosylation, and we have not yet clarified how NgBR deficiency affects the trafficking of lipoproteins and other proteins through hypoglycosylation. Future research should establish a quantitative and reproducible liver and plasma glycoproteomics approach to quantify specific protein glycosylation. In addition, we observed a 40% penetrance of HCC development in D-LKO mice, not the anticipated 100% penetrance seen with NgBR loss. Further investigation is needed to understand this, possibly by measuring hepatic dolichol or protein glycosylation levels. Additionally, our analysis of NgBR protein levels was conducted in patients with MASLD/MASH, not in those with MASH-derived HCC, due to challenges in obtaining clinical liver samples from MASH-derived HCC patients, which are often mixed with virus- or alcohol-induced HCC cases.

## Methods

### Sex as a biological variable.

Experiments were conducted exclusively in male mice. Male mice were used because they are more susceptible to HFD-induced obesity and show more consistent weight gain, enabling a robust and reproducible model. Female mice are relatively resistant to diet‑induced weight gain, which can influence metabolic outcomes. Notably, we observed a similar phenotype, including HCC development in N-LKO mice, in female mice under diet‑induced obese conditions. Although our findings are based on male mice, the underlying mechanisms are expected to be relevant across sexes; nonetheless, future studies including females are needed to confirm sex‑specific effects and generalizability.

### Animal studies.

Mice bearing a loxP-flanked NgBR allele (*NgBR^loxP/loxP^* mice) or Dhdds allele (*Dhdds^loxP/loxP^* mice) were generated as described previously (8, 48). Liver-specific NgBR- or Dhdds-knockout mice (N-LKO or D-LKO) were generated by breeding albumin-Cre *NgBR^loxP/+^* or albumin-Cre *Dhdds^loxP/+^* mice with *NgBR^loxP/+^* or *Dhdds^loxP/+^* mice, respectively. All mouse strains were in the C57BL6 genetic background. *N-LKO* or *Dhdds* mice were confirmed for NgBR or Dhdds KO in the liver by PCR using Cre primers and primers flanking the 5′ homology arm of the NgBR gene or Dhdds gene and LoxP sites from the tail-extracted DNA. Heterozygous NgBR R294H mutant mice were created on the C57BL6 background by the knockin technology at Yale University. PCR primers were designed that covered the targeted region (forward primer, 5′-TCTAGGCTCTGTCACCCGCA-3′, and reverse primer, 5′-TCTAGGCTCTGTCACCCGCA-3′), amplifying a 269 bp fragment of DNA in both WT and NgBR R294H mice. The knockin sequence was confirmed by restriction enzyme digestion with BStN1, which cleaves the knockin allele.

All experimental mice were housed in a barrier animal facility with a constant temperature and humidity in a 12-hour dark/12-hour light cycle while water and food were provided ad libitum. All mice (*n* = 3–5 per cage) were fed with a standard CD for 8 weeks, after which they were switched to an HFD (60% calories from fat; D12492, Research Diets) or a high-fat, high-cholesterol WD containing 1.25% cholesterol (D12108, Research Diets) for 4 months to induce HCC. Mice used in all experiments were sex- and age-matched and kept in ventilated cages in a pathogen-free facility.

### Lipoprotein profile and lipid measurements.

To analyze the plasma samples, blood was collected from overnight-fasted mice via the tail vein, and plasma was separated by centrifugation at 9,600*g* at 40°C for 10 minutes. Plasma TAGs were enzymatically analyzed with commercially available kits (Wako Pure Chemicals). The distribution of lipoproteins in the plasma lipid fractions was analyzed by FPLC gel filtration using 2 Superose 6 HR 10/30 columns (Pharmacia Biotech).

### Hepatic VLDL-TAG secretion.

To measure the liver VLDL-TAG secretion rate, mice were fasted overnight and administered an intraperitoneal injection of 1 g/kg of body weight Poloxamer 407 (Sigma-Aldrich) dissolved in PBS. Blood samples were collected from the tail immediately before the injection and at 1, 2, 3, and 4 hours after injection, following previously described methods (49). The circulating TAG levels were analyzed using commercially available kits.

### Fat tolerance test.

A fat tolerance test was conducted following a previously established protocol (49). In summary, mice underwent a 4-hour fasting period, after which they received an oral gavage of 10 μL of olive oil per gram of body weight. Blood samples were then collected from the tail vein at 0, 1, 2, and 4 hours after the administration of olive oil. Plasma TAG levels were measured at the specified time intervals, as previously described (49).

### Tissue lipid uptake.

To assess lipid uptake in the tissues, we employed a previously described method using ^3^H-labeled triolein (49). In brief, mice underwent a 4-hour fasting period and were orally administered a 100 μL emulsion containing 2 μCi of [^3^H]-triolein. After a 2-hour interval, we harvested the organs and extracted lipids from each tissue using a mixture of isopropyl alcohol and hexane (in a 2:3 ratio). Subsequently, we separated the lipid layer and quantified the radioactivity of [^3^H]-triolein (measured in cpm) using liquid scintillation counting in a beta counter.

### Assessment of HCC tumor nodule burden and volume.

Following euthanasia, livers from N-LKO mice fed an HFD or WD were harvested. Liver images were captured from both anterior and posterior views with a ruler included for scale. Visible HCC nodules were identified, and tumor volume was calculated using the following formula: volume = 0.52 × (width)² × (length).

### Liver histology and lipid measurement.

The liver samples were analyzed by preparing them for histological examination and lipid content determination. For histological analysis, the liver tissues were fixed in 4% paraformaldehyde, sectioned, processed into paraffin blocks, and stained with H&E. To visualize neutral lipids, frozen liver samples were embedded in OCT, sectioned, and stained with Oil Red O using the Oil Red O staining method. The total TAG levels were extracted using a chloroform/methanol solvent (2:1) based on a previously described method (49), and the liver TAG content was measured using a commercially available assay kit (Sekisui Diagnostics) according to the manufacturer’s instructions.

### ALT, AST, and AFP measurements.

ALT and AST activity and plasma AFP levels were determined in plasma with commercially available assay kits (MAK052 and MAK055, Sigma-Aldrich) following manufacturer’s recommendations.

### Circulating leukocyte analysis.

Blood was collected via the tail vein in heparinized microhematocrit capillary tubes, and the total numbers of circulating blood leukocytes were analyzed using the HEMAVET system (Drew Scientific). For further characterization of leukocytes, FACS analysis was performed as follows. Erythrocytes were lysed using ACK lysis buffer (155 mM ammonium chloride, 10 mM potassium bicarbonate, and 0.01 mM EDTA, pH 7.4), and leukocytes were blocked with 2 μg/mL of FcgRII/III. The leukocytes were then stained with a mixture of antibodies, and monocytes were identified as CD115^hi^ and subsets as Ly6-C^hi^ and Ly6-C^lo^. Neutrophils were identified as CD11b^hi^Ly6G^hi^, B cells were identified as CD19^hi^B220^hi^, and T cells were identified as CD4^hi^ or CD8^hi^. The following antibodies were used for all leukocyte analyses (all from BioLegend): FITC-Ly6-C (HK1.4), PE-CD115 (AFS98), APC-Ly6-G (1A8), PB-CD11b (M1/70), APC-CD19 (6D5), PE/Cy7-B220 (RA3-6B2), APC/Cy7-CD4 (RM4-5), and BV421-CD8a (53-6.7). All antibodies were used at 1:300 dilutions.

### Isolation of primary hepatocytes and NPCs and hepatic immune cell flow cytometry analysis.

Hepatic cells were isolated from the liver of both WT and N-LKO mice obtained from the Yale Liver Center, as previously described (multistep mechanism of polarized Ca^2+^ wave patterns in hepatocytes) (50). Briefly, mice were anesthetized with isoflurane and attached to a Styrofoam tray. The abdomen was wet with 70% ethanol and opened along the midline, and a ligature was placed around the mid-portal vein and inferior vena cava. The portal vein was cannulated with a 22 G catheter and perfused with Hanks’ A followed by Hanks’ B with collagenase. After the liver was removed, hepatocytes were released by shaking, and the cell suspension was filtered through a 40 μm mesh. The cells were then pelleted by centrifugation, and the supernatant was aspirated. The cell pellet was resuspended in L-15 media for further use. The suspension was subjected to centrifugation at 60*g* for 2 minutes to separate pellets containing hepatocytes and NPCs. NPCs, which include immune cells, were isolated by centrifuging the suspension at 300*g* for 5 minutes and resuspension in 200 μL of ACK solution (155 mM ammonium chloride, 10 mM potassium bicarbonate, and 0.01 mM EDTA, pH 7.4). The NPCs were then stained with a mixture of antibodies to identify specific cell types. B cells were identified using APC-Cy7 B220 (BioLegend), while T cells were identified using CD4^hi^ or CD8^hi^ with the following antibodies: PE/Cy7 CD45.2 (100830, BioLegend), APC/Cy7 CD4 (100525, BioLegend), and BV605 CD8a (100744, BioLegend). Macrophages were identified using Pacific blue (123124, BioLegend), and neutrophils were identified using Apc/Cy7 CD11b (1012245, BioLegend) and APC Ly6G (127614, BioLegend). All antibodies were used at a 1:300 dilution.

### Cis-PTase assay.

To assay cis-PTase activity in mammalian cells, a reaction mixture was prepared consisting of 250 μg microsomal protein, 25 μM FPP, 50 μM [1-^14^C]-isopentenyl pyrophosphate (IPP) (55 mCi/mmol), 25 mM Tris-HCl (pH 7.4), 1 mM MgCl_2_, 1.25 mM DTT, 2.5 mM sodium orthovanadate, 10 μM zaragozic acid A, and 0.35% Triton X-100 in a total volume of 0.1 mL. The reaction was performed at 37°C for 2 hours and terminated by the addition of 10 μL of concentrated hydrochloric acid. The polyprenol diphosphates were chemically dephosphorylated by incubating the lipids at 90°C for 1 hour. The products were extracted with 4 mL of chloroform/methanol (3:2) and washed 3 times with one-fifth volume of 10 mM EDTA in 0.9% NaCl. The chloroform was evaporated under a stream of nitrogen, and the dephosphorylated lipids were loaded onto HPTLC RP-18 precoated plates and run in acetone containing 50 mM H_3_P0_4_. The plates were then exposed to film to visualize the products of IPP incorporation. To measure the incorporation of radioactive IPP into the polyprenol fraction, the gel from the zone containing radiolabeled polyprenols was scraped and subjected to liquid scintillation counting. All reagents used were of analytical grade and purchased from Sigma-Aldrich and Thermo Fisher Scientific; [1-^14^C] IPP (50 mCi/mmol) was purchased from American Radiolabeled Chemicals. The reverse-phase TLC (HPTLC RP-18) plates used were from MilliporeSigma (catalog 1.16225.0001).

### Western blot analysis.

To prepare the liver homogenates, we utilized the Bullet Blender Homogenizer method as previously described (49). Tissues were lysed in an ice-cold buffer containing 50 mM Tris-HCl (pH 7.5), 0.1% SDS, 0.1 mM EDTA, 0.1% deoxycholic acid, 0.1 mM EGTA, 1% NP-40, 5.3 mM NaF, 1.5 mM Na_4_P_2_O_7_, 1 mM orthovanadate, 1 mg/mL protease inhibitor cocktail (Roche), and 0.25 mg/mL AEBSF (Roche). The lysates were sonicated and rotated at 4°C for 1 hour, followed by centrifugation at 12,000*g* for 30 minutes.

After normalizing the protein concentration, equal amounts of proteins were resuspended in SDS sample buffer and separated by SDS-PAGE. The separated proteins were then transferred onto nitrocellulose membranes and probed with various antibodies, such as anti-ATF4 (Cell Signaling, 11815, 1:1,000), anti-NgBR (Abcam, Ab168351), anti–β-actin (Sigma-Aldrich, A1978), and anti-HSP90 (F-8) (Santa Cruz Biotechnology, sc-13119). The protein bands were detected using the Odyssey Infrared Imaging System (LI-COR Biotechnology), and densitometry was performed using ImageJ (NIH) software.

For the immunoblot analysis of ApoB-100 and ApoB-48 in pooled VLDL lipoprotein fractions, separation was performed using a NuPAGE Novex 3%–15% Tris-Acetate Mini Gel with 1× NuPAGE Tris-Acetate SDS running buffer (Invitrogen). Following an overnight transfer of proteins onto nitrocellulose membranes, the membranes were blocked with nonfat milk dissolved in 5% (w/v) wash buffer. The membrane was then probed with an antibody against ApoB (Meridian, K23300R, 1:2,000) overnight at 4°C.

### Comparative gene expression profiling in healthy human liver and liver with MASH.

To investigate the expression of Nus1 in human liver with nonalcoholic steatohepatitis (MASH) compared with healthy liver, we obtained RNA-seq gene expression data from a publicly available human liver cohort (GSE135251) through the Gene Expression Omnibus (GEO). Processed data files were downloaded from GEO (data processing is outlined in ref. 51), then normalized and transformed using edgeR-limma-voom as described (52, 53). The results are presented as box-and-whisker plots, with the central lines representing medians, the edges of the box indicating upper and lower quartiles, and the whiskers indicating the minimum and maximum values.

### Cell lines and culture conditions.

We acquired the human liver cell line Huh7 (01042712 from Sigma-Aldrich). These cells were cultured in DMEM supplemented with 2 mM/L glutamine and 10% FBS, along with penicillin/streptomycin (Life Technologies). HEK293T (ATCC, CRL-3216) cells were also part of our study and were cultured in DMEM high glucose supplemented with 10% FBS and 1% penicillin/streptomycin. We regularly screened these cells for mycoplasma contamination to maintain their integrity.

### Dolichol-P supplementation in hepatocyte cultures.

Primary hepatocytes isolated from chow-fed N-LKO mice were cultured for 24 hours under 3 conditions: serum-free medium with 0.2% BSA plus dolichol-P (50 μg/mL), serum-free medium with 0.2% BSA alone, or medium containing 10% FBS. Dolichyl monophosphate was a gift from Ewa Kula-Świezewska and Karolina Skorupińska-Tudek (Institute of Biochemistry and Biophysics, Polish Academy of Sciences, Warsaw, Poland).

### Transfection with plasmids and siRNA.

For stable NgBR knockdown in Huh7 cells, lentiviral-mediated transduction was employed (54). Specific shRNAs targeting NgBR were used, and cells were subsequently selected in puromycin following a previously established protocol. Additionally, a transient knockdown experiment was conducted by transfecting siRNAs against NgBR (Dharmacon) into Huh7 cells using RNAiMAX (Life Technologies) following the manufacturer’s instructions. The efficiency of knockdown was assessed by quantifying NgBR expression through RT-qPCR.

### RNA isolation and RT-qPCR.

The isolation of total RNA from tissue or cells was conducted using TRIzol reagent (Invitrogen) in accordance with the manufacturer’s instructions. Subsequently, cDNA was synthesized using iScript RT Supermix (Bio-Rad) as per the manufacturer’s protocol for mRNA expression analysis. For RT-qPCR analysis, Sso Fast Eva Green Supermix (Bio-Rad) was utilized, and the measurements were performed on an iCycler Real-Time Detection System (Eppendorf). The mRNA levels were normalized to 18S.

### Measurement of ROS generation.

The cellular ROS species H_2_O_2_ and O_2_^–^ were analyzed in primary hepatocytes from WT and N-LKO mice using DCFDA or DHE dye, following the manufacturer’s instructions (Thermo Fisher Scientific). First, isolated hepatocytes were incubated with 5 μM DCFDA or DHE dye for 30 minutes at 37°C. Next, the cells were washed twice with PBS, and their fluorescence was acquired using flow cytometry (FACSAria, BD Biosciences).

### Deep RNA-seq.

Total RNA was isolated and purified from both the livers of control and N-LKO mice or tumors of N-LKO mice using an RNA isolation kit from Qiagen, followed by DNAse treatment to eliminate any genomic contamination using RNA Min Elute Cleanup from Qiagen. The purity of the total RNA samples was confirmed using the Agilent Bioanalyzer from Agilent Technologies. The RNA-seq was performed by the Yale Center for Genome Analysis after the removal of rRNA from RNA samples using the Ribo-Zero rRNA Removal Kit from Illumina. The RNA libraries were created using the TrueSeq Small RNA Library preparation kit (Illumina) and then sequenced for 45 cycles on the Illumina HiSeq 2000 platform (1 × 75 bp read length). To ensure high quality of the reads, scripts developed in-house were used to trim for quality, and the reads were aligned to the reference genome using TopHat2. Transcript abundances and differences were calculated using cuffdiff, and the results were analyzed using R and cummeRbund through scripts developed in-house.

### scRNA-seq and data analysis.

We performed scRNA-seq on livers using the 10x Genomics Chromium platform. Hepatic cells were isolated from both WT and N-LKO mice obtained from the Yale Liver Facility Center. We used a multistep mechanism for isolating hepatocytes from the liver, which has been described previously (50). Briefly, the mice were anesthetized with isoflurane and attached to a Styrofoam tray. The abdomen was wet with 70% ethanol, and a ligature was placed around the mid-portal vein and inferior vena cava. The portal vein was cannulated with a 22 G catheter and perfused with Hanks’ A followed by Hanks’ B with collagenase. The liver was then removed, and the hepatocytes were released by shaking. The cell suspension was filtered through a 40 μm mesh, and the cells were pelleted by centrifugation. The cell pellet was resuspended in L-15 media for further use.

We mixed hepatocytes and NPCs in a 1:1 ratio for WT mice and hepatocytes, NPCs, and tumor cells in a 1:1:0.25 ratio for N-LKO mice. We loaded single cells onto the 10x Genomics Chromium Single-Cell controller at the Yale Center for Genome Analysis, followed by lysis and barcoded reverse transcription of polyadenylated mRNA from each cell using the Single Cell 3′ Reagent Kit v3 (10x Genomics). The libraries were sequenced on an Illumina HiSeq 4000 as 2 × 150 paired-end reads.

We used Cell Ranger software for sample demultiplexing, read alignment, and unique molecular identifier processing. Low-quality cells, doublets, and potentially dead cells were filtered out based on the percentage of mitochondrial genes and number of genes and unique molecular identifiers expressed in each cell.

To cluster the cells, we used the Seurat R package with filtered genes by barcode expression matrices as inputs. We calculated highly variable genes using the Seurat function Find Variable Genes and used them for downstream clustering analysis. We visualized clustering of liver single-cell transcriptomes (7,000 cells from WT and 7,000 cells from N-LKO mice fed WD) using UMAP. UMAP was performed with the Run UMAP function (Seurat) using highly variable genes for dimensionality reduction. Clustering was done through the Find Clusters function using 30 significant principal components with a resolution of 0.3. We also identified significantly differentially expressed genes in a cluster using the Seurat function Find All Markers; these genes were expressed in more than 25% of cells with at least 0.25-fold difference, reaching statistical significance of an adjusted *P* < 0.05, as determined by Wilcoxon’s test.

### Statistics.

The mouse sample size for each study was based on literature documentation of similar well-characterized experiments. The number of mice used in each experiment is listed in the figure legends. No inclusion or exclusion criteria were used, and studies were not blinded to investigators or formally randomized. Data are expressed as mean ± SEM. Statistical differences were measured using an unpaired 2-sided Student’s *t* test or 1- or 2-way ANOVA with Bonferroni’s correction for multiple comparisons. Normality was tested using the Kolmogorov-Smirnov test. A nonparametric test (Mann-Whitney) was used when the data did not pass the normality test. A value of *P* ≤ 0.05 was considered statistically significant. Data analysis was performed using GraphPad Prism software.

### Study approval.

All animal procedures were conducted in accordance with protocols reviewed and approved by the Yale University School of Medicine Institutional Animal Care and Use Committee.

### Data availability.

The RNA-seq and scRNA-seq data have been deposited in GEO (GSE230972). All other data generated in this study are provided in the main text and in the supplemental materials. Values for all data points in graphs are provided in the Supporting Data Values file.

## Author contributions

AKS and WCS conceived and designed the study, and wrote the manuscript. AKS conducted the majority of the experiments and analyzed the data. BC performed experiments, analyzed the scRNA-seq data, and contributed to manuscript editing. KMC and JWMF analyzed the deep RNA-seq data. KH isolated the liver cells from the mice. MS and JC conducted the animal experiments. IRM ran the FPLC for lipoprotein profiling. KAG measured the cis-PTase enzyme activity. SL and SCL conducted experiments and analyzed the data. SS and NEB conducted experiments, analyzed data, and contributed to manuscript editing. SAM designed the glycosylation study. SCL and KEM performed the experiments. TTR provided the DGAT2 inhibitor. JK analyzed the data. YS and CFH supervised KMC and IRM in conducting the experiments and data analysis.

## Conflict of interest

The authors have declared that no conflict of interest exists.

## Funding support

This work is the result of NIH funding, in whole or in part, and is subject to the NIH Public Access Policy. Through acceptance of this federal funding, the NIH has been given a right to make the work publicly available in PubMed Central.

- NIH grants R35HL139945 and RO1DK125492 (to WCS).
- NIH grant K01DK124441 (to NEB).
- American Heart Association MERIT Award (to WCS).
- American Heart Association Postdoctoral Fellowship Award (to SL).
- NIH award P30 DK034989 to Yale Liver Center Cellular and Molecular Physiology Core.

## Supplementary Material

Supplemental data

Unedited blot and gel images

Supporting data values

## Version 1 03/10/2026

In-Press Preview

## Version 2 05/01/2026

Electronic publication

## References

[1] El-Serag HB (2011). Hepatocellular carcinoma. N Engl J Med.

[2] Garrido A, Djouder N (2021). Cirrhosis: a questioned risk factor for hepatocellular carcinoma. Trends Cancer.

[3] White DL (2012). Association between nonalcoholic fatty liver disease and risk for hepatocellular cancer, based on systematic review. Clin Gastroenterol Hepatol.

[4] Browning JD, Horton JD (2004). Molecular mediators of hepatic steatosis and liver injury. J Clin Invest.

[5] Michelotti GA (2013). NAFLD, NASH and liver cancer. Nat Rev Gastroenterol Hepatol.

[6] Gallage S (2022). A researcher’s guide to preclinical mouse NASH models. Nat Metab.

[7] Park EJ (2016). NgBR is essential for endothelial cell glycosylation and vascular development. EMBO Rep.

[8] Park EJ (2014). Mutation of Nogo-B receptor, a subunit of cis-prenyltransferase, causes a congenital disorder of glycosylation. Cell Metab.

[9] Edani BH (2020). Structural elucidation of the *cis*-prenyltransferase NgBR/DHDDS complex reveals insights in regulation of protein glycosylation. Proc Natl Acad Sci U S A.

[10] Grabinska KA (2016). cis-Prenyltransferase: new insights into protein glycosylation, rubber synthesis, and human diseases. J Biol Chem.

[11] Galosi S (2022). De novo DHDDS variants cause a neurodevelopmental and neurodegenerative disorder with myoclonus. Brain.

[12] Zelinger L (2011). A missense mutation in DHDDS, encoding dehydrodolichyl diphosphate synthase, is associated with autosomal-recessive retinitis pigmentosa in Ashkenazi Jews. Am J Hum Genet.

[13] Wen R (2013). Aberrant dolichol chain lengths as biomarkers for retinitis pigmentosa caused by impaired dolichol biosynthesis. J Lipid Res.

[14] Turner GA (1992). N-glycosylation of serum proteins in disease and its investigation using lectins. Clin Chim Acta.

[15] DelaCourt A (2021). N-glycosylation patterns correlate with hepatocellular carcinoma genetic subtypes. Mol Cancer Res.

[16] Costa AF (2020). Targeting glycosylation: a new road for cancer drug discovery. Trends Cancer.

[17] Starosta RT (2021). Liver manifestations in a cohort of 39 patients with congenital disorders of glycosylation: pin-pointing the characteristics of liver injury and proposing recommendations for follow-up. Orphanet J Rare Dis.

[18] Marques-da-Silva D (2017). Liver involvement in congenital disorders of glycosylation (CDG). A systematic review of the literature. J Inherit Metab Dis.

[19] Lipinski P (2021). Liver involvement in congenital disorders of glycosylation and deglycosylation. Front Pediatr.

[20] Tahata S (2023). Liver transplantation recovers hepatic N-glycosylation with persistent IgG glycosylation abnormalities: three-year follow-up in a patient with phosphomannomutase-2-congenital disorder of glycosylation. Mol Genet Metab.

[21] Iancu TC (2007). The liver in congenital disorders of glycosylation: ultrastructural features. Ultrastruct Pathol.

[22] Wilson MP (2024). A pseudoautosomal glycosylation disorder prompts the revision of dolichol biosynthesis. Cell.

[23] Eggens I (1989). Polyisoprenoid, cholesterol and ubiquinone levels in human hepatocellular carcinomas. Br J Exp Pathol.

[24] Stender S (2017). Adiposity amplifies the genetic risk of fatty liver disease conferred by multiple loci. Nat Genet.

[25] Fabbrini E (2010). Obesity and nonalcoholic fatty liver disease: biochemical, metabolic, and clinical implications. Hepatology.

[26] Anstee QM (2019). From NASH to HCC: current concepts and future challenges. Nat Rev Gastroenterol Hepatol.

[27] Hu W (2016). Nogo-B receptor deficiency increases liver X receptor alpha nuclear translocation and hepatic lipogenesis through an adenosine monophosphate-activated protein kinase alpha-dependent pathway. Hepatology.

[28] Chen Y (2021). NGBR is required to ameliorate type 2 diabetes in mice by enhancing insulin sensitivity. J Biol Chem.

[29] Pfister D (2021). NASH limits anti-tumour surveillance in immunotherapy-treated HCC. Nature.

[30] Yuan ZY (2019). Serum amyloid A levels in patients with liver diseases. World J Gastroenterol.

[31] Hendrikx T (2022). Soluble TREM2 levels reflect the recruitment and expansion of TREM2^+^ macrophages that localize to fibrotic areas and limit NASH. J Hepatol.

[32] Busch CJ (2017). Malondialdehyde epitopes are sterile mediators of hepatic inflammation in hypercholesterolemic mice. Hepatology.

[33] Poli G (1987). The role of lipid peroxidation in liver damage. Chem Phys Lipids.

[34] Hotamisligil GS (2010). Endoplasmic reticulum stress and the inflammatory basis of metabolic disease. Cell.

[35] Keenan RW (1976). The binding of [3H]dolichol by plasma high density lipoproteins. Biochim Biophys Acta.

[36] Ginsberg HN, Mani A (2022). Complex regulation of fatty liver disease. Science.

[37] Donnelly KL (2005). Sources of fatty acids stored in liver and secreted via lipoproteins in patients with nonalcoholic fatty liver disease. J Clin Invest.

[38] Amin NB (2019). Targeting diacylglycerol acyltransferase 2 for the treatment of nonalcoholic steatohepatitis. Sci Transl Med.

[39] Futatsugi K (2015). Discovery and optimization of imidazopyridine-based inhibitors of diacylglycerol acyltransferase 2 (DGAT2). J Med Chem.

[40] Wolf MJ (2014). Metabolic activation of intrahepatic CD8+ T cells and NKT cells causes nonalcoholic steatohepatitis and liver cancer via cross-talk with hepatocytes. Cancer Cell.

[41] Asgharpour A (2016). A diet-induced animal model of non-alcoholic fatty liver disease and hepatocellular cancer. J Hepatol.

[42] Kishida N (2016). Development of a novel mouse model of hepatocellular carcinoma with nonalcoholic steatohepatitis using a high-fat, choline-deficient diet and intraperitoneal injection of diethylnitrosamine. BMC Gastroenterol.

[43] Guri Y (2017). mTORC2 promotes tumorigenesis via lipid synthesis. Cancer Cell.

[44] Horie Y (2004). Hepatocyte-specific Pten deficiency results in steatohepatitis and hepatocellular carcinomas. J Clin Invest.

[45] Nakagawa H (2014). ER stress cooperates with hypernutrition to trigger TNF-dependent spontaneous HCC development. Cancer Cell.

[46] Park EJ (2010). Dietary and genetic obesity promote liver inflammation and tumorigenesis by enhancing IL-6 and TNF expression. Cell.

[47] Tsuchida T (2018). A simple diet- and chemical-induced murine NASH model with rapid progression of steatohepatitis, fibrosis and liver cancer. J Hepatol.

[48] Ramachandra Rao S (2020). Retinal degeneration caused by rod-specific dhdds ablation occurs without concomitant inhibition of protein N-glycosylation. iScience.

[49] Singh AK (2021). Hepatocyte-specific suppression of ANGPTL4 improves obesity-associated diabetes and mitigates atherosclerosis in mice. J Clin Invest.

[50] Nathanson MH (1994). Multistep mechanism of polarized Ca2+ wave patterns in hepatocytes. Am J Physiol.

[51] Govaere O (2020). Transcriptomic profiling across the nonalcoholic fatty liver disease spectrum reveals gene signatures for steatohepatitis and fibrosis. Sci Transl Med.

[52] Law CW (2014). voom: precision weights unlock linear model analysis tools for RNA-seq read counts. Genome Biol.

[53] Law CW (2016). RNA-seq analysis is easy as 1-2-3 with limma, Glimma and edgeR. F1000Res.

[54] Tiscornia G (2006). Design and cloning of lentiviral vectors expressing small interfering RNAs. Nat Protoc.

