# Supplementary material for: Impaired glycosylation promotes rapid transition to hepatocellular carcinoma in model of diet-induced steatotic liver disease
Source: J Clin Invest. 2026 Mar 10;136(9):e197719. doi: 10.1172/JCI197719 (PMC13132402; doi:10.1172/JCI197719)
Supplement: Supplemental data [file jci-136-197719-s335.pdf]

# Impaired glycosylation promotes rapid transition to hepatocellular carcinoma in model of diet-induced steatotic liver disease

Abhishek K. Singh[1*], Balkrishna Chaube[2,3], Kathryn M Citrin[2], Joseph Wayne Fowler[1], Sungwoon Lee[1], Jonatas Catarino[4], James Knight[5], Sarah Lowery[6], Sonal Shree[7], Keira E. Mahoney[6], Nabil Boutagy[1], Inmaculada Ruz-Maldonado[2], Kathy Harry[8], Marya Shanabrough[4], Trenton Thomas Ross[9], Stacy A. Malaker[6], Yajaira Suárez[2,10] Carlos Fernández-Hernando[2,10], Kariona A Grabińska[1] and William C. Sessa[1*]

[1]Department of Pharmacology, and Vascular Biology and Therapeutics Program, Yale University School of Medicine, New Haven, Connecticut, 06520, USA.

[2]Department of Comparative Medicine, Yale Center for Molecular and Systems Metabolism and Vascular Biology and Therapeutics Program, Yale University School of Medicine, New Haven, CT, USA.

[3]Department of Biosciences and Bioengineering, Indian Institute of Technology Dharwad, Dharwad, Karnataka, India 580007

[4]Integrative Cell Signaling and Neurobiology of Metabolism Program, Department of Comparative Medicine, Yale University School of Medicine, New Haven, CT, USA

[5]Department of Genetics, Yale School of Medicine, New Haven, CT, USA

[6]Department of Chemistry, Yale University, New Haven, CT, USA

[7]Molecular Biophysics & Biochemistry, Yale University, New Haven, CT, USA

[8]Department of Internal Medicine, Yale University, New Haven, CT, USA

[9]Internal Medicine Research Unit (IMRU), Pfizer, Cambridge, MA, USA

[10]Department of Pathology, Yale University School of Medicine, New Haven, CT, USA

***Corresponding authors:**

Address Correspond to : Abhishek K. Singh email: abhishek.singh@yale.edu and William C. Sessa, email: william.sessa@yale.edu, Vascular Biology & Therapeutics Program, Department

of Pharmacology, Yale University School of Medicine, Amistad Research Building, 10 Amistad St, New Haven, CT 06520, USA, Tel: (203)737-2291.

**Conflict of Interest:** The authors declare no competing interests.

## Supplemental information titles and legends

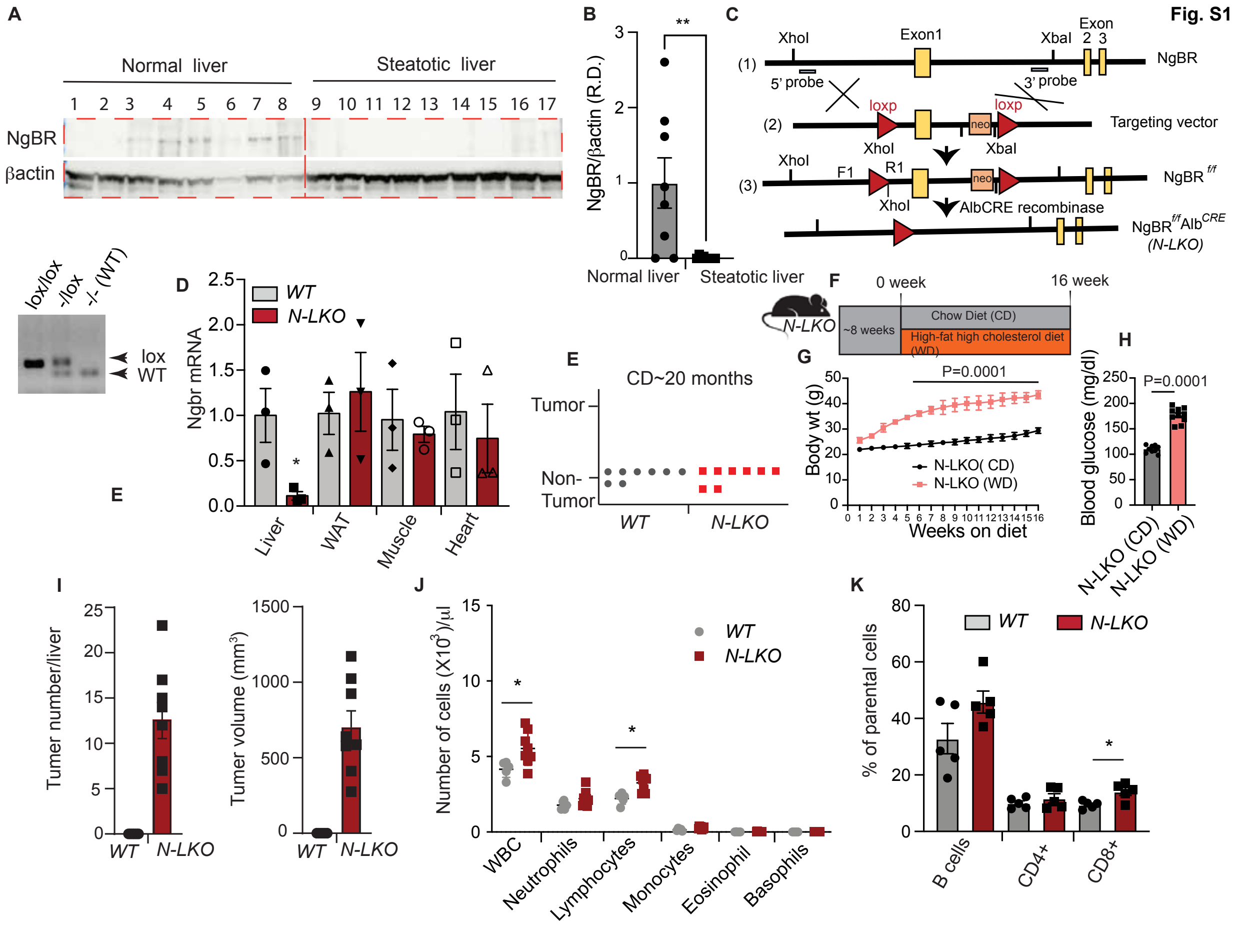


**Figure S1 Hepatic NgBR depletion promotes HCC formation in obesity-induced models and aligns with MASLD-MASH progression in human liver. (A)** Immunoblot images depicting NgBR and β-actin protein expression in human liver tissue samples (1-8 Normal liver samples and 9-17 steatotic liver). **Table 1-** Tabulated information summarizing the disease profiles of patients with liver conditions **(B)** Densitometric analysis results are presented in the corresponding right panels. R.D. represents relative density. Two-sided P < 0.01, comparing steatotic liver disease patients with healthy controls using an unpaired Welch's t-test. **(C)** Schematic diagram showing the generation of liver-specific NgBR knockout (N-LKO) mice. (1) The NgBR genomic DNA fragment is composed of three exon 1-3 (2) Schematic construct of the NgBR targeting vector. NgBR exon1 is flanked by two loxP sites. (3) Mice with floxed allele are generated after homologous recombination. Consequently, these mice were bred with mice expressing CRE recombinase to generate tissue-specific NgBR-knockout mice. Right panel represents PCR amplification of $NgBR^{f/f}$ mice showing bands from both, one, or none of the floxed alleles. **(D)** mRNA expression of NgBR in the liver, WAT, heart and muscle of WT and N-LKO mice (n=3). NgBR expression in these tissues is normalized to its expression in liver from WT mice. **(E)** The graph show no tumor incidence in N-LKO mice fed an CD for ~ 20 months. **(F)** Outline of the developmental timeline of obesity-induced HCC in N-LKO mice fed with a High-Fat Cholesterol Diet (WD). **(G)** Body weight of N-LKO mice fed CD or WD for 16 weeks. **(H)** Fasting blood glucose was measured in N-LKO mice fed CD or a WD after 16 weeks. **(I)** Graph showing hepatic tumor number and volume in N-LKO mice after 16 weeks of WD feeding (n=8) **(J)**

Peripheral blood cell counts from WT and N-LKO mice fed HFD for 16 weeks, measured using a hemavet hematology analyzer (n=5). **(K)** Flow cytometry analysis of circulating B and T cells (n=5). Each data point represents a biological replicate. $P < 0.05$; $P < 0.001$ by unpaired Welch's t-test, and $P < 0.001$ by two-way ANOVA with Sidak's multiple comparisons test, comparing N-LKO with WT mice.

**Table1** Liver disease profiles of patients

| | |
|---|---|
| 9 | Steatohepatitis |
| 10 | Steatosis |
| 11 | Steatosis |
| 12 | Steatosis, micro and macrovesicular |
| 13 | Steatosis, mild |
| 14 | Steatosis, mild |
| 15 | Steatosis, mild |
| 16 | Steatosis, mild, periventricular and bridging fibrosis |
| 17 | Steatosis, sporadic foamy hepatocytes, hemophagocytic syndrome |

Fig. S2

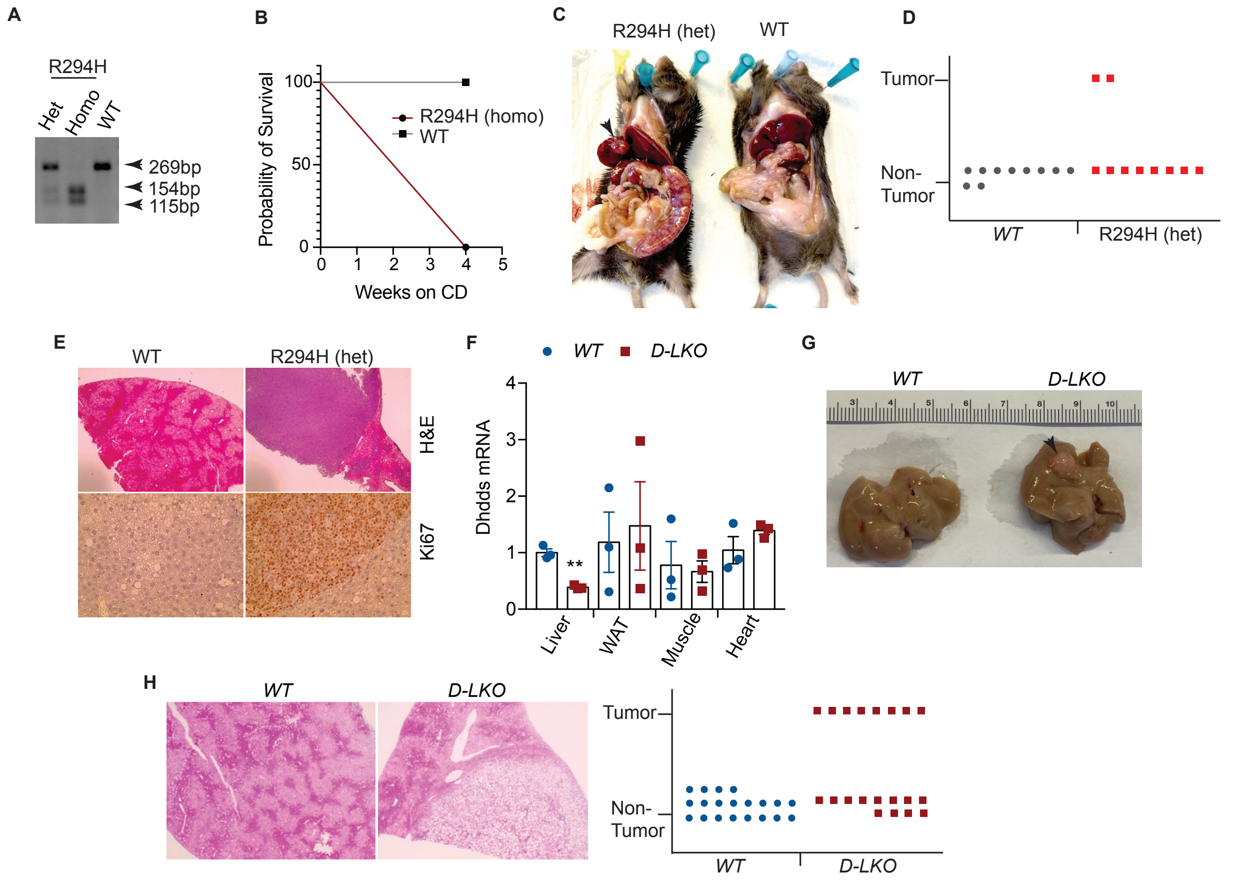


**Figure S2. Mutation in NgBR or depletion of Dhdds in the liver drives the HCC development in HFD- diet conditions. (A)** Generation of NgBR R-294H mutant mice. PCR and agarose gel electrophoresis of WT, R294H point mutant homozygous and heterozygous tail samples. Genotyping from *NgBR R294H mutant* mice presentation bands from one (WT,269bp) two (homozygous-154+115bp) or three (heterozygous -269 and 154+115bp). **(B)** Kaplan-Meier survival curves of WT and R294H mutant homozygous mice. **(C)** Representative image of mice with liver cancer of R294H heterozygous mutant and WT fed HFD for 16 weeks. arrow showing toward HCC. **(D)** Right panel shows graph summarizing R294H mutant heterozygous and WT mice with or without tumor on HFD (n=8). Symbols display individual mice. **(E)** Histological analysis of liver and tumor sections stained with H&E and Ki-67 isolated from WT and R294H mutant heterozygous mice on HFD. **(F)** mRNA expression of Dhdds in the liver, WAT, muscle, and heart of WT and D-LKO mice (n=3). Dhdds expression in the tissue (s) is normalized to its expression in liver from WT mice. **(G)** Representative images of the liver isolated from WT and D-LKO mice fed a HFD for 16 weeks, arrow showing toward HCC. Right panel represents histological analysis of liver and tumor sections stained with H&E and right panel shows graph summarizing D-LKO and WT mice with or without tumor feeding on HFD. Symbols represent individual mice. Scale bar, 200 μm. Two-sided; $^{**}P < 0.01$; comparing *D-LKO* with WT mice using an unpaired Welch's t-test.

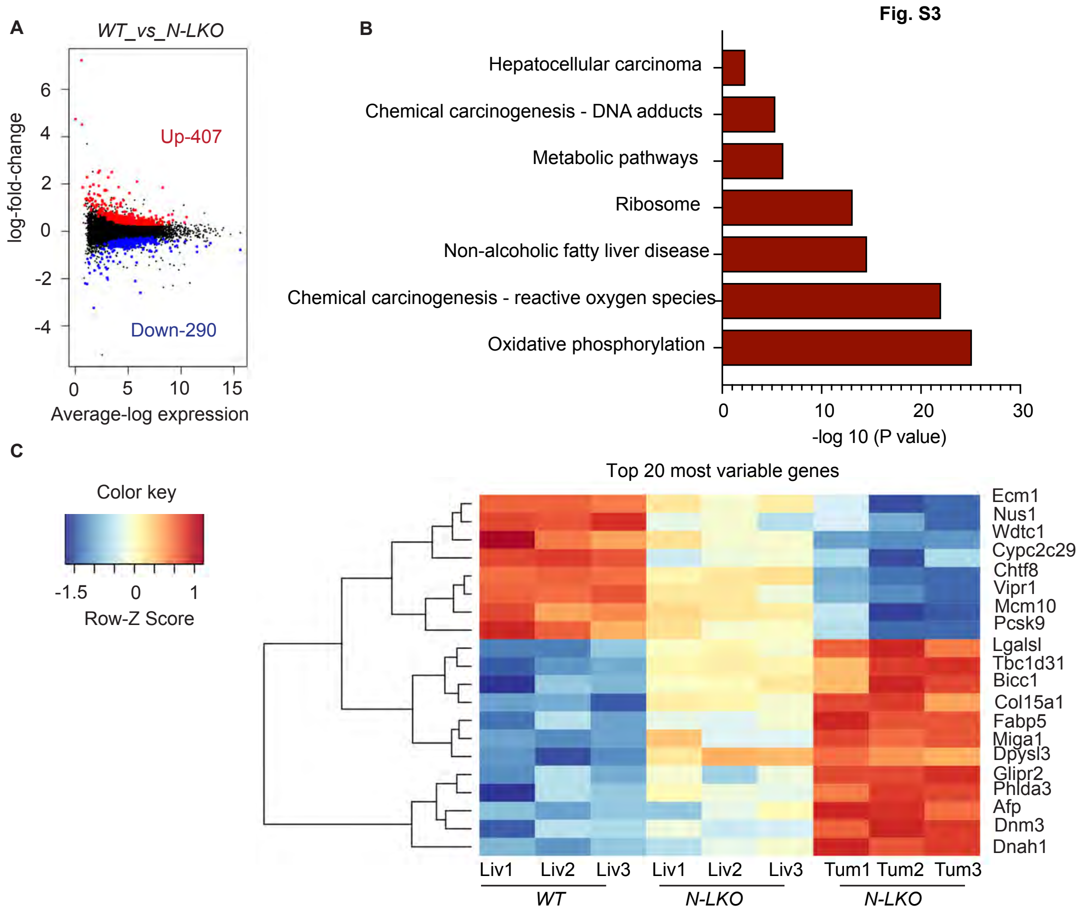


**Figure S3. Liver-specific NgBR deficiency enhances the expression of genes associated with MASLD and HCC.**

**(A)** Bulk RNA sequencing was performed on liver tissues from WT and liver-specific NgBR knockout (N-LKO) mice fed a high-fat diet (HFD) for 16 weeks. A multidimensional scaling (MDS) plot illustrates distinct clustering of gene expression profiles between WT and N-LKO groups, indicating genotype-driven transcriptional divergence. Differential expression analysis revealed that hepatic NgBR deficiency led to the upregulation of 407 genes and downregulation of 290 genes (n=4), many of which are implicated in lipid metabolism, oxidative stress, and tumorigenesis. **(B)** KEGG pathway enrichment analysis of the upregulated genes in N-LKO livers (n=3) identified significant activation of pathways involved in steatotic liver formation, reactive oxygen species (ROS) production, DNA damage response, and oncogenic signaling, suggesting a mechanistic link between NgBR loss and hepatocellular carcinoma (HCC) development.

**(C)** RNA sequencing of liver tissues and tumor samples from N-LKO mice fed a Western diet (WD) for 16 weeks (n=3) revealed a distinct gene expression signature in tumor tissues. The heatmap highlights upregulation of HCC-associated markers such as alpha-fetoprotein (AFP) and fatty acid-binding protein 5 (FABP5), alongside downregulation of PCSK9, a gene involved in cholesterol metabolism. These transcriptional changes underscore the role of NgBR deficiency in driving hepatic carcinogenesis under lipid-rich dietary conditions.

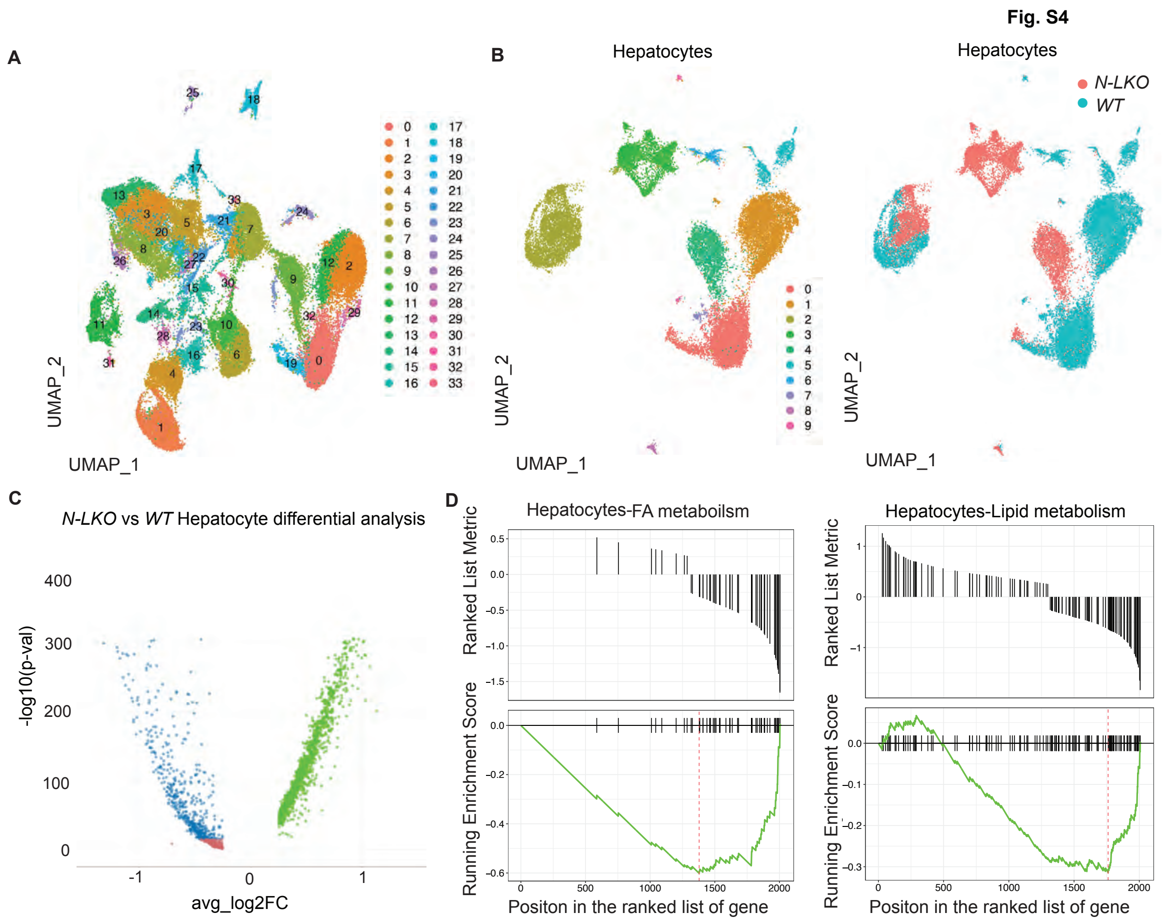


**Figure S4. Liver-specific NgBR ablation induces distinct and differential gene expression patterns in liver cells. (A)** Single-cell RNA sequencing analysis of liver cells from WT and N-LKO mice fed a Western diet (WD) UMAP plot showing 33 distinct clusters of cells isolated from the liver of WT and N-LKO mice fed WD. **(B)** UMAP plots representing 9 subclusters of hepatocytes in WT and N-LKO mice. The color represents the subcluster or genotype. **(C)** Volcano plot displaying the differential expression of genes in log-transformed upregulated and downregulated genes. **(D)** Gene set enrichment analysis (GSEA) plot representing the downregulation of genes in hepatocytes involved in fatty acid and lipid metabolism.

Fig. S5

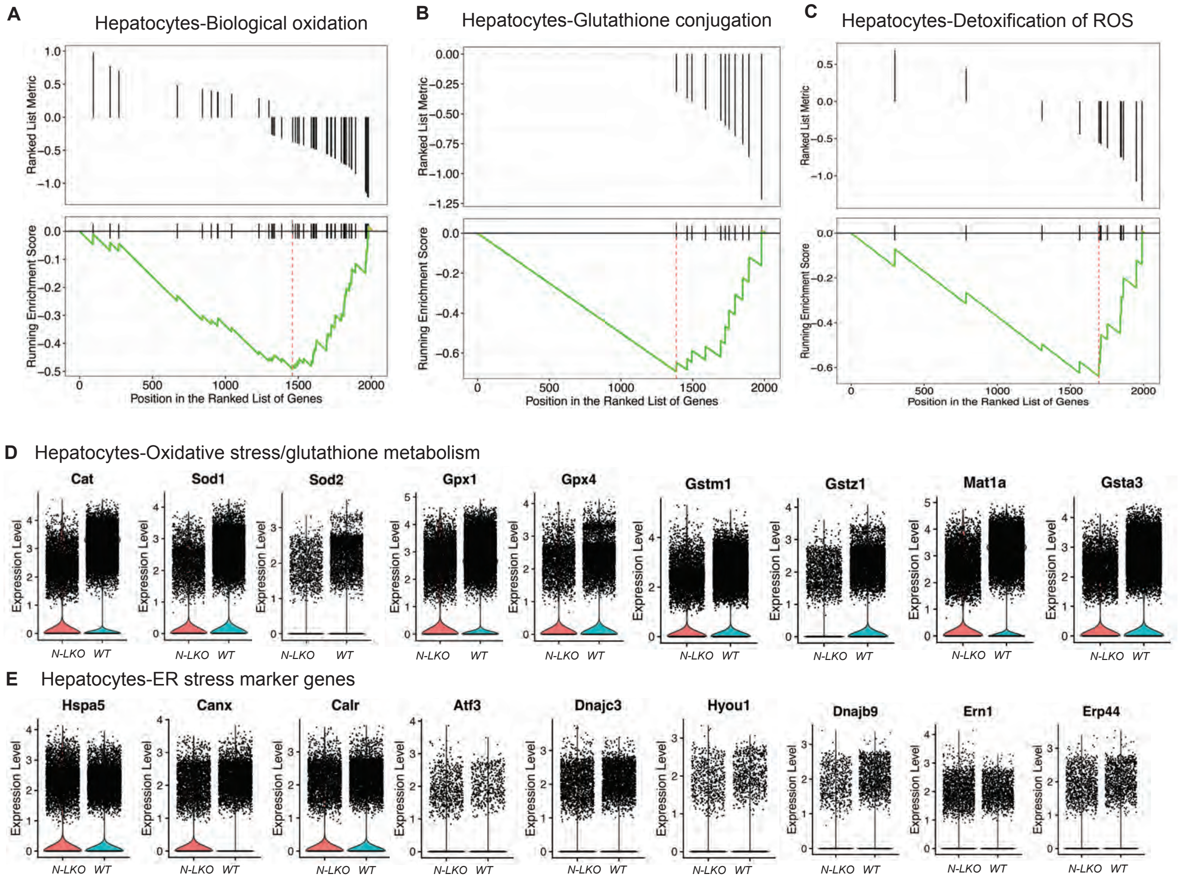


**Figure S5. Liver-specific NgBR depletion downregulates oxidative stress response in hepatocytes.** Single-cell RNA sequencing analysis of hepatic cells from WT and N-LKO mice fed a Western diet (WD**). (A-C)** Gene set enrichment analysis (GSEA) plot representing the downregulation of genes involved in biological oxidation, glutathione conjugation and detoxification of ROS (reactive oxygen species) in hepatocytes of N-LKO mice compared to those of WT mice. (**D-E)** The violin plots indicate a significant reduction in the expression of genes related to oxidative stress, glutathione metabolism, and ER stress in the hepatocytes of N-LKO compared to WT mice.

Fig. S6

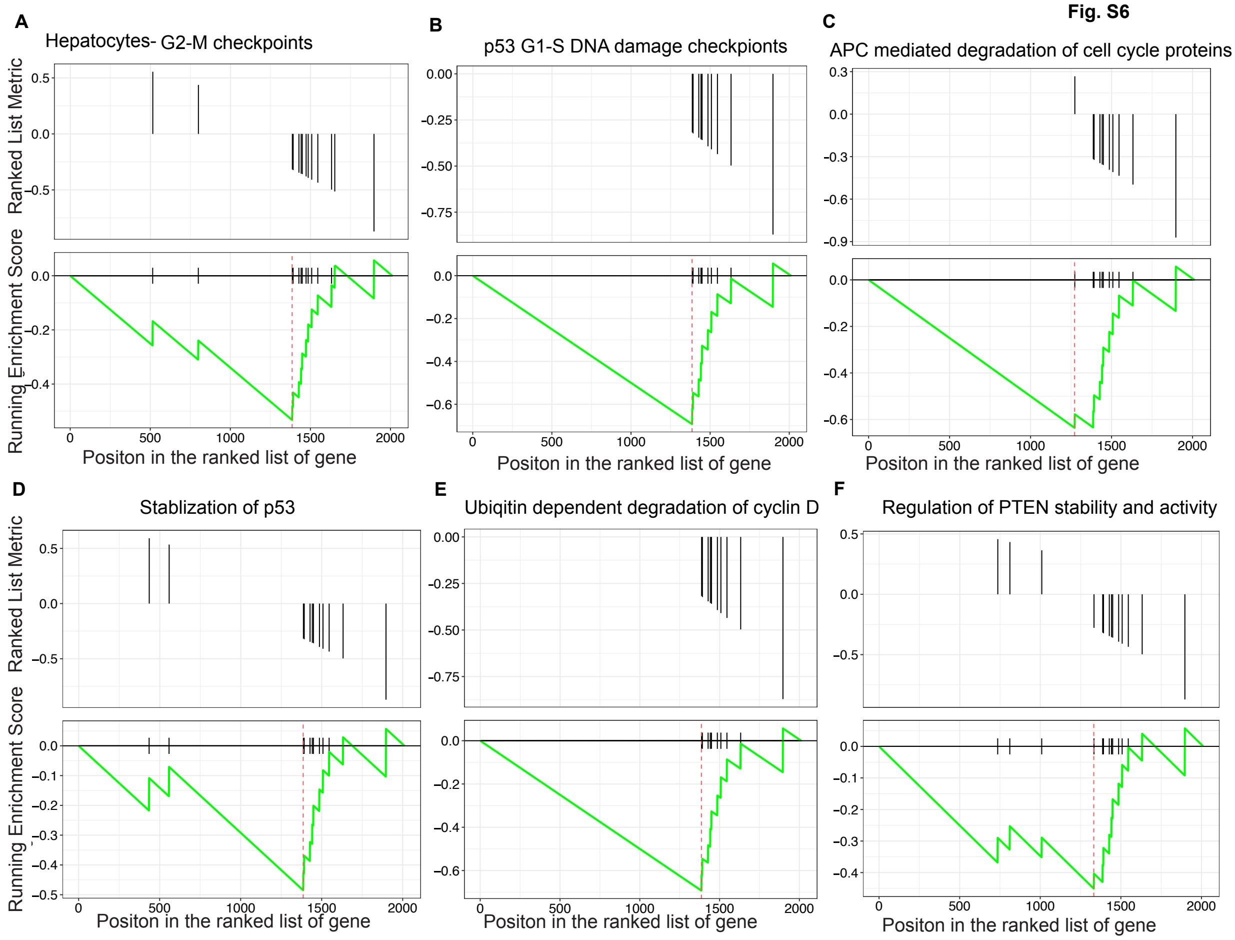


**Figure S6. Lack of hepatic NgBR reduces the cell cycle checkpoint regulators in hepatocytes.** Single-cell RNA seq analysis distinct gene expression patterns in the hepatocytes of WT and N-LKO mice fed a Western diet. **(A)** Gene set enrichment analysis (GSEA) plot representing genes involved in the G2-M checkpoint regulation are downregulated in the hepatocytes of N-LKO mice compared to WT mice. **(B)** Genes involved in p53-dependent G1-S DNA damage checkpoint regulation are significantly downregulated in N-LKO mice compared to WT mice fed a WD. **(C)** APC-mediated degradation of cell cycle proteins: genes involved in the APC-mediated degradation of cell cycle proteins are downregulated in the N-LKO mice compared to WT mice. **(D)** Stabilization of p53: genes involved in the stabilization of p53, a tumor suppressor protein that regulates the cell cycle, are downregulated in the hepatocyte of N-LKO mice compared to WT mice. **(E)** Ubiquitin-dependent degradation of cyclin D: genes involved in the ubiquitin-dependent degradation of cyclin D, a protein that regulates the G1 phase of the cell cycle, are downregulated in the hepatocyte of N-LKO mice compared to WT mice. **(F)** Regulation of PTEN stability and activity: genes involved in the regulation of PTEN stability and activity, a tumor suppressor protein that regulates cell cycle progression and cell growth, are downregulated in the hepatocytes of N-LKO mice compared to WT mice. The normalized enrichment score (NES) for each gene set is shown on the plot, with negative NES indicating downregulation of genes in the N-LKO mice compared to WT mice.

**Fig. S7**

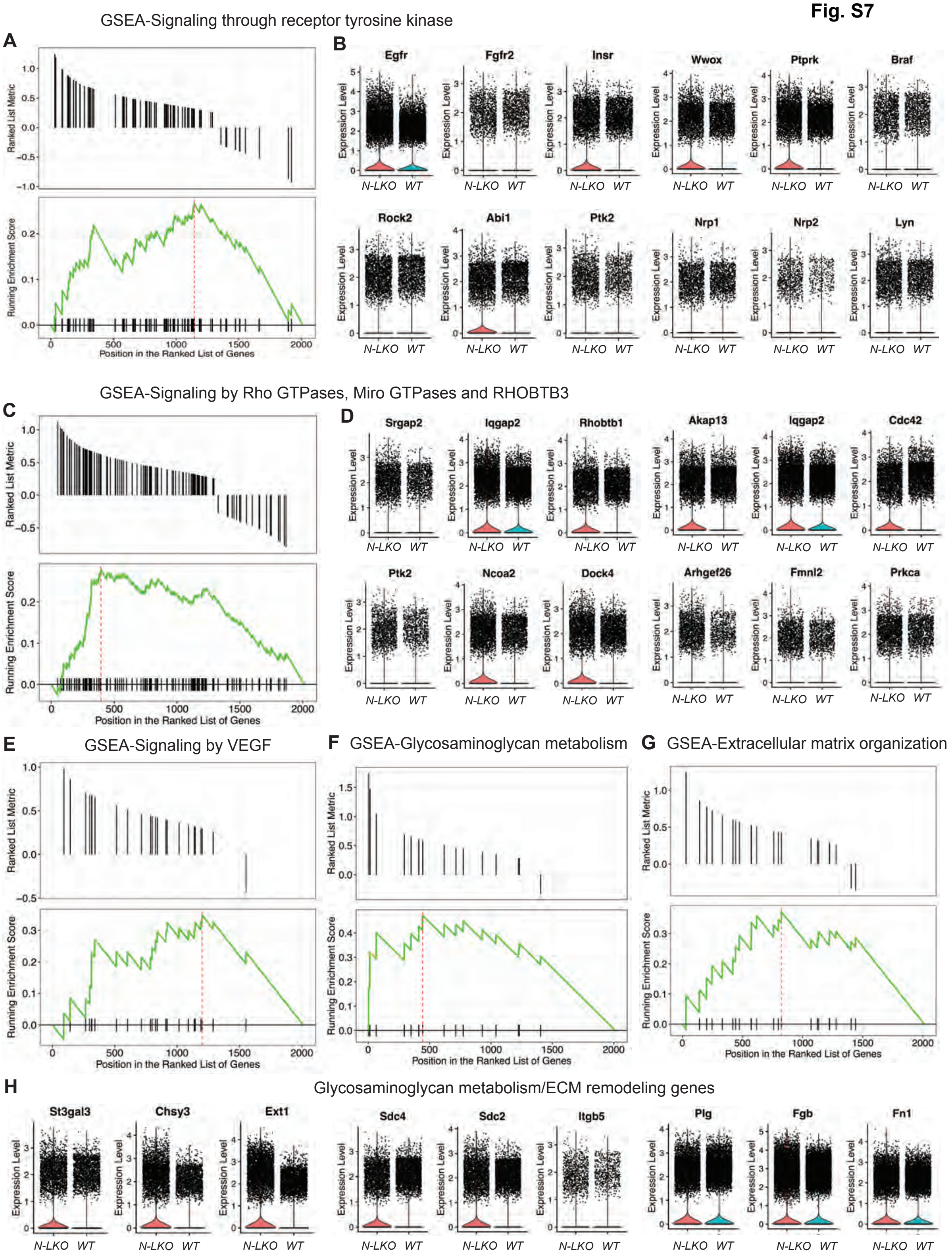

A
GSEA-Signaling through receptor tyrosine kinase
Ranked List Metric
Running Enrichment Score
Position in the Ranked List of Genes
B
Egfr
Fgfr2
Insr
Wwox
Ptprk
Braf
Rock2
Abi1
Ptk2
Nrp1
Nrp2
Lyn
Expression Level
N-LKO
WT
C
GSEA-Signaling by Rho GTPases, Miro GTPases and RHOBTB3
D
Srgap2
Iqgap2
Rhobtb1
Akap13
Iqgap2
Cdc42
Ptk2
Ncoa2
Dock4
Arhgef26
Fmnl2
Prkca
E
GSEA-Signaling by VEGF
F
GSEA-Glycosaminoglycan metabolism
G
GSEA-Extracellular matrix organization
H
Glycosaminoglycan metabolism/ECM remodeling genes
St3gal3
Chsy3
Ext1
Sdc4
Sdc2
Itgb5
Plg
Fgb
Fn1

**Figure S7. Absence of NgBR in hepatocytes promotes oncogenic associated pathway.** Single-cell RNA sequencing analysis that identified distinct gene expression patterns in the hepatocytes of N-LKO mice that were fed a Western diet. **(A)** The gene set enrichment analysis (GSEA) plot reveals that genes involved in signaling via tyrosine receptors were upregulated in hepatocytes of N-LKO mice compared to WT mice. **(B)** The violin plots further reveal significantly increased expression levels of hepatocyte genes involved in activation of tyrosine receptor signaling in N-LKO mice compared to WT mice. **(C)** The GSEA plot shows the upregulation of genes involved in Rac/Rho GTPase signaling in hepatocytes of N-LKO mice compared to WT mice. **(D)** The violin plots illustrate expression levels of hepatocyte genes involved in Rac/Rho GTPase signaling were significantly elevated in N-LKO mice relative to WT mice. **(E-G)** The GSEA plots show the upregulation of genes involved in VEGF signaling, glucosamine glycans metabolism, and ECM organization in hepatocytes of N-LKO mice compared to WT mice. **(H)** The violin plots demonstrate a significantly increase in the expression of genes involved in glucosamine glycans metabolism and ECM remodeling in N-LKO mice relative to WT mice.

Fig. S8

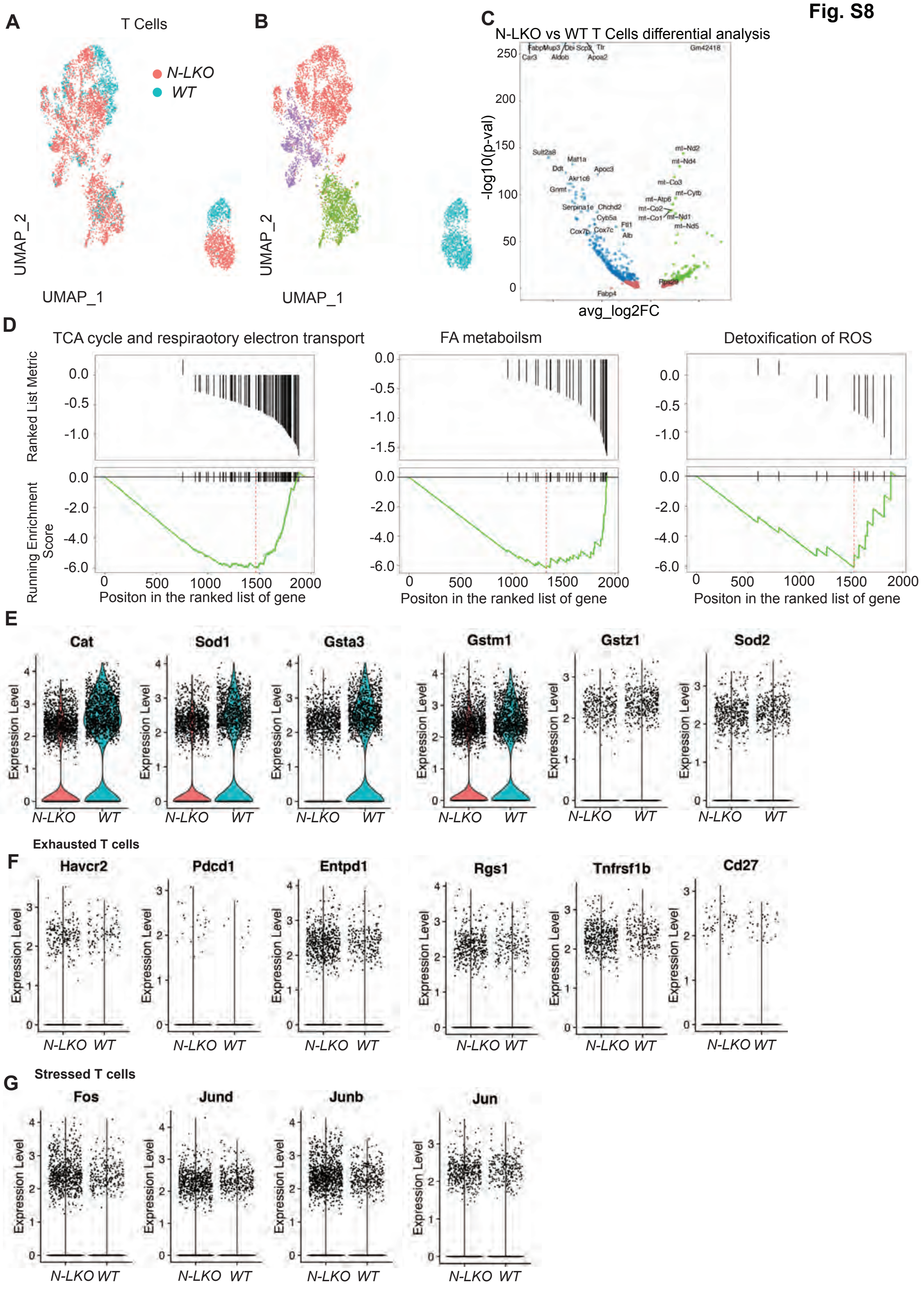


**Figure S8 Hepatic NgBR loss alters T cell expression profiles associated with exhaustion.** The single-cell RNA sequencing analysis of liver cells from WT and N-LKO mice fed a WD revealed important differences in the gene expression patterns of T cells. **(A-B)** The UMAP plot

displays four distinct subclusters of T cells from both groups, colored according to genotype. The color represents the subcluster or genotype. **(C)** The Volcano plot illustrates the differential expression of genes in upregulated and downregulated T cells in N-LKO mice compared to WT mice. **(D)** GSEA plot indicates the downregulation of genes involved in mitochondrial respiratory function, fatty acid metabolism, and detoxification of ROS in T cells of N-LKO mice compared to WT mice. **(E)** The violin plots demonstrate that T cells from N-LKO mice had significantly downregulated antioxidant genes compared to those from WT mice. **(F-G)** The Violin plots show a significant upregulation of genes associated with T cell exhaustion and stress in hepatic T cells of N-LKO mice compared to WT mice.

**Fig. S9**

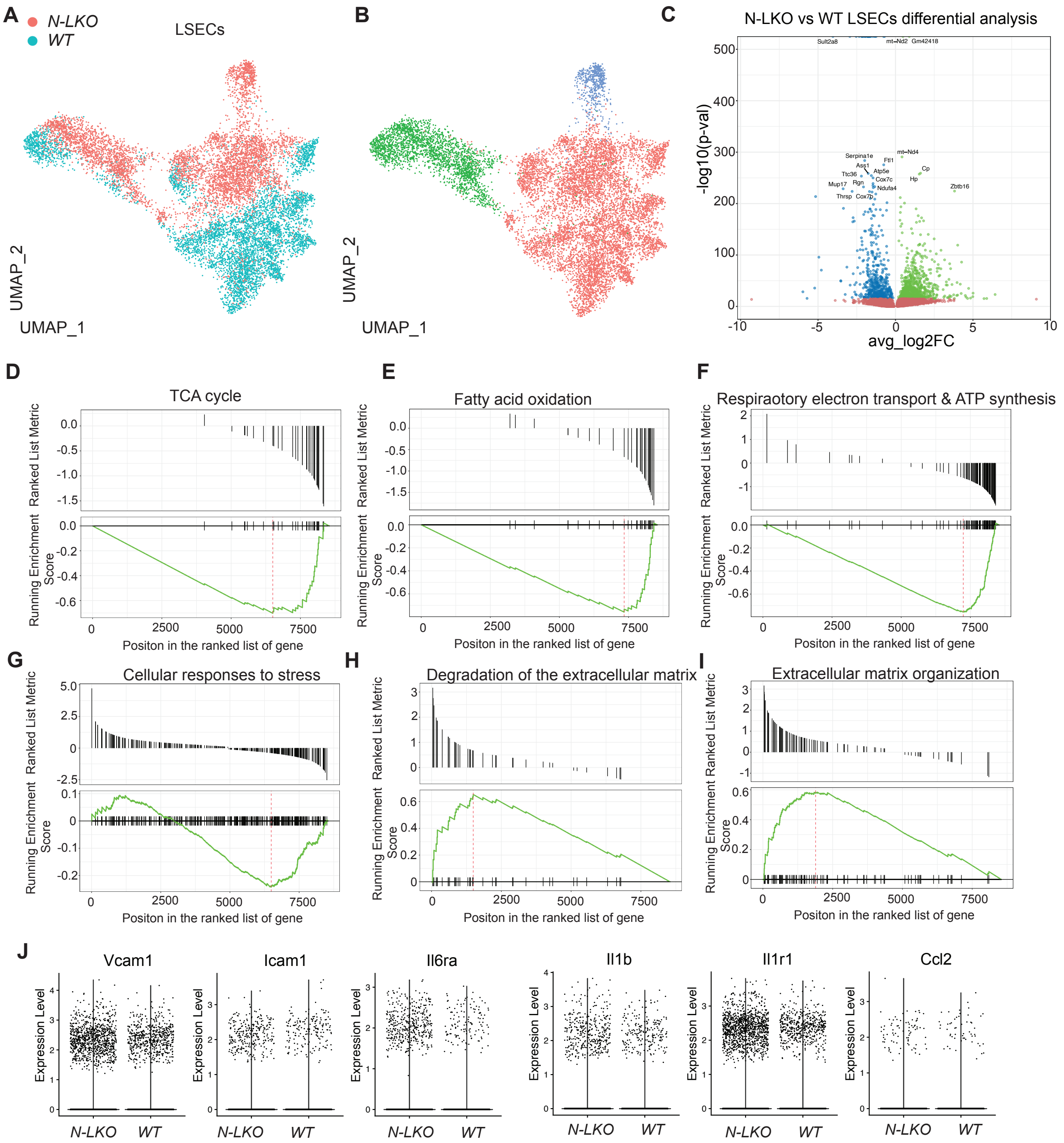

A
N-LKO
WT
LSECs
UMAP_2
UMAP_1
B
UMAP_2
UMAP_1
C
N-LKO vs WT LSECs differential analysis
-log10(p-val)
avg_log2FC
Sult2a8
mt-Nd2
Gm42418
Serpina1e
mt-Nd4
Ftl1
Ass1
Atp5e
Cp
Ttc36
Cox7c
Hp
Mup17
Rgn
Ndufa4
Zbtb16
Thrsp
Cox7b
D
TCA cycle
Ranked List Metric
Running Enrichment Score
Positon in the ranked list of gene
E
Fatty acid oxidation
Ranked List Metric
Running Enrichment Score
Positon in the ranked list of gene
F
Respiraotory electron transport & ATP synthesis
Ranked List Metric
Running Enrichment Score
Positon in the ranked list of gene
G
Cellular responses to stress
Ranked List Metric
Running Enrichment Score
Positon in the ranked list of gene
H
Degradation of the extracellular matrix
Ranked List Metric
Running Enrichment Score
Positon in the ranked list of gene
I
Extracellular matrix organization
Ranked List Metric
Running Enrichment Score
Positon in the ranked list of gene
J
Vcam1
Icam1
Il6ra
Il1b
Il1r1
Ccl2
Expression Level
N-LKO
WT

**Figure S9. Hepatic loss of NgBR leads to endothelial dysfunction.** Single-cell RNA sequencing of liver cells from (WT and N-LKO mice fed a WD revealed significant alterations in gene expression within liver sinusoidal endothelial cells (LSECs). **(A–B)** UMAP plots show three distinct LSEC subclusters, colored by genotype and subcluster identity, highlighting transcriptional differences between WT and N-LKO groups. **(C)** Volcano plot displays differentially expressed genes in LSECs from N-LKO mice compared to WT, indicating both upregulated and downregulated transcripts. **(D-F**) Gene Set Enrichment Analysis (GSEA) reveals downregulation of pathways related to oxidative metabolism, including the tricarboxylic acid (TCA) cycle, fatty acid oxidation, respiratory electron transport, ATP synthesis, and cellular stress responses in N-LKO LSECs. **(G-I)** GSEA also shows upregulation of genes associated with extracellular matrix remodeling in N-LKO LSECs. **(J)** Violin plots demonstrate significantly increased expression of inflammatory genes in LSECs from N-LKO mice compared to WT.

**Fig. S10**

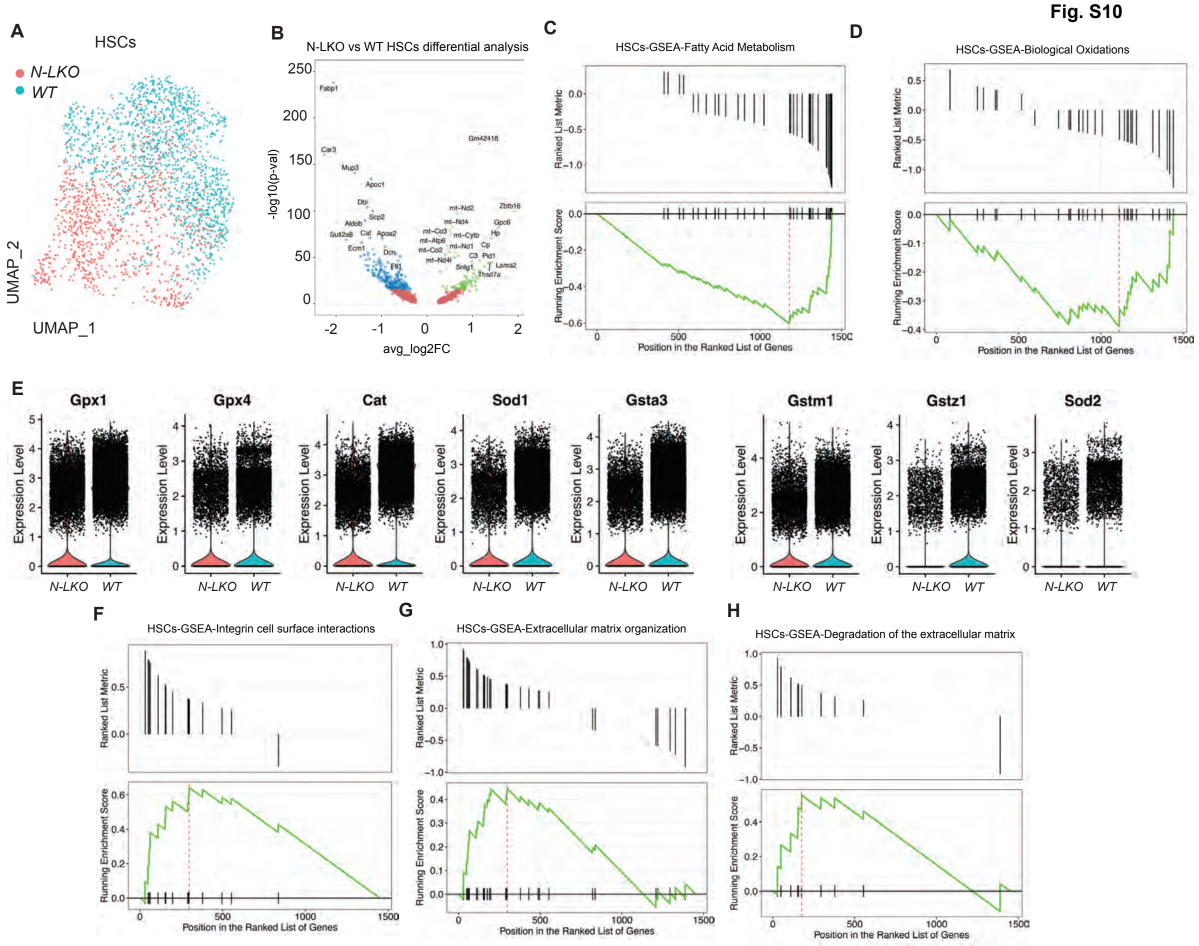


**Figure S10. Loss of NgBR in hepatocytes triggers the activation of hepatic stellate cell gene profile.** Impact of hepatic loss of NgBR on the hepatic stellate cell gene profile, as analyzed through single-cell RNA sequencing of liver cells from WT and N-LKO mice fed a Western diet (WD). **(A)** The UMAP plot illustrates distinctive subclusters of hepatic stellate cells in both groups, which are distinguished by color. **(B)** The volcano plots illustrate the differential expression of genes, demonstrating significant upregulation and downregulation of hepatic stellate cells in N-

LKO mice compared to WT mice. **(C-D)** Gene Set Enrichment Analysis (GSEA) plots that show the downregulation of HSC genes involved in lipid oxidation in N-LKO compared to WT mice **(E)** The violin plots visually depicted the significant downregulation of expression levels of HSC genes involved in the oxidative stress response in N-LKO mice relative to WT mice. **(F-H)** GSEA plots that show upregulation of HSC genes on extracellular cell matrix (ECM) remodeling.

**Fig. S11**

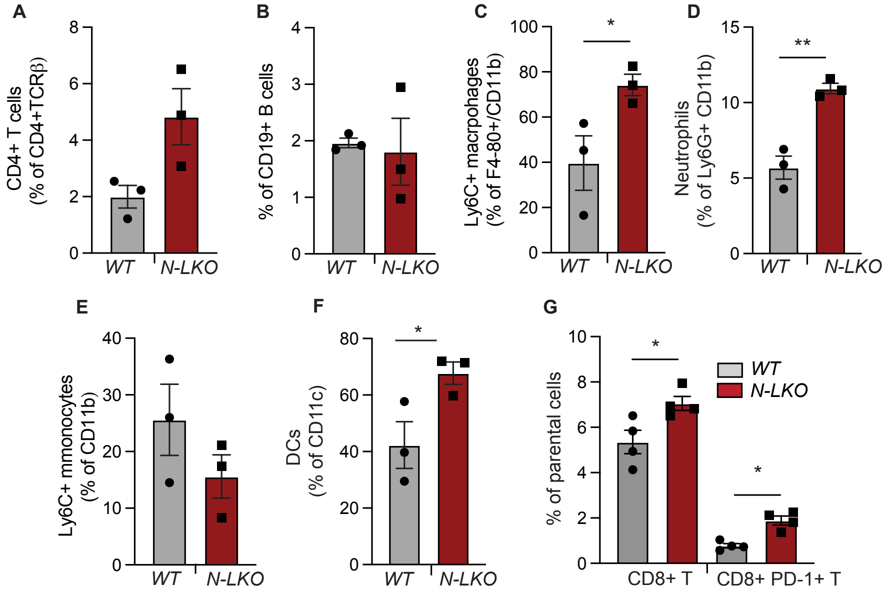


**Figure S11. Lack of NgBR in the liver enhances hepatic immune infiltration.**
Flow cytometric analysis was performed on hepatic immune cell populations isolated from WT and liver-specific NgBR knockout (N-LKO) mice after 16 weeks of high-fat diet (HFD) feeding. Both lymphoid and myeloid subsets were evaluated. **(A–B)** Among lymphoid cells, $CD4^+$ T cells were increased in N-LKO mice, but the change did not reach statistical significance, while $CD19^+$ B cells showed no notable difference. **(C-F)** Within the myeloid compartment, N-LKO mice exhibited elevated levels of macrophages, neutrophils, and dendritic cells (DCs), whereas monocyte levels remained unchanged. **(G)** Immunosuppressive $CD8^+PD\text{-}1^+$ T cells were significantly increased in N-LKO mice, indicating enhanced T cell exhaustion a hallmark of chronic liver inflammation. Statistical significance was determined using a two-sided unpaired Welch's t-test; *P < 0.05, **P < 0.01.

Fig. S12

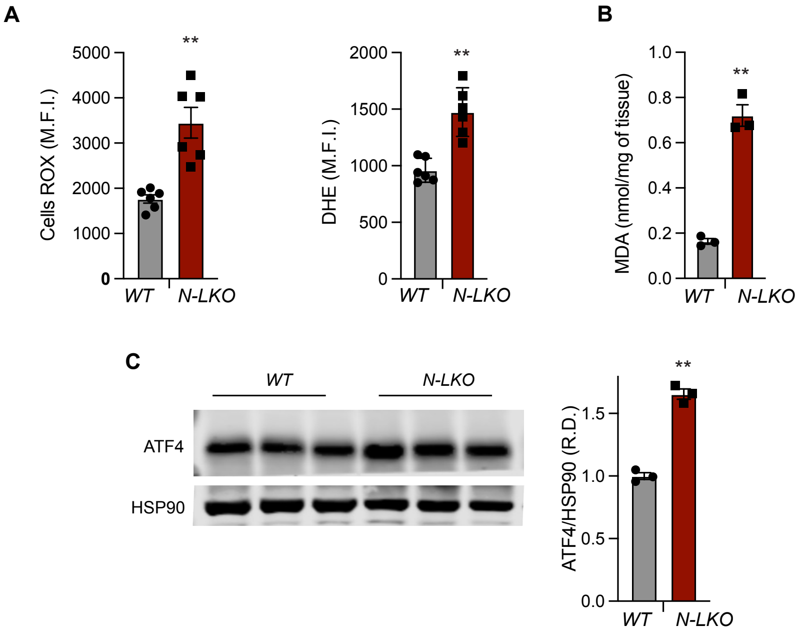


**Figure S12. Absence of NgBR in the liver induces oxidative and ER stress under diet-induced obese conditions.**
**(A)** Analysis of cellular ROS production in the primary hepatocytes isolated from WT, N-LKO fed a HFD (n=6). **(B)** Membrane lipid peroxidation determined via MDA assay in the liver isolated from WT and N-LKO fed HFD (n=3). **(C)** Representative immunoblot blot and densiometric analysis of an ER key stress response proteins ATF4 and housekeeping standard HSP90 in the liver isolated from WT and N-LKO fed HFD (n=3).Two-sided ***P* < 0.01; comparing N-LKO with WT mice using an unpaired Welch's t-test.

Fig. S13

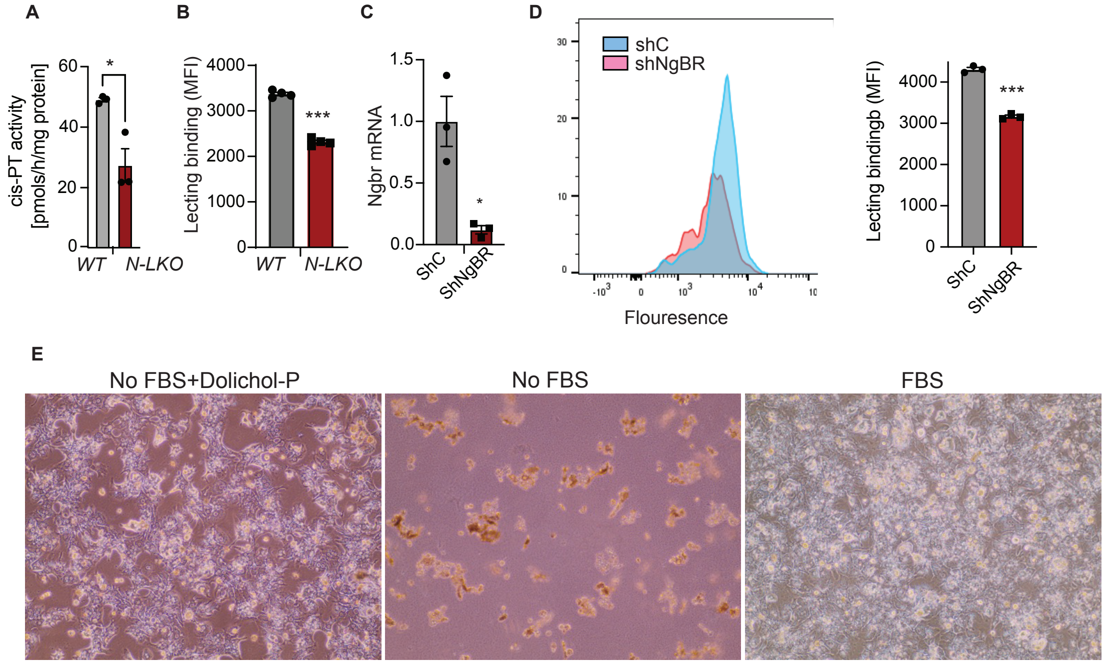


**Figure S13. The Loss of NgBR function in the hepatocyte reduces cis-PTase enzyme activity and protein glycosylation, leading to impaired hepatocyte survival in a dolichol-dependent manner. (A)** Measurement of microsomal Cis-PTase activity in isolated hepatocytes of WT and N-LKO mice (n=3). **(B)** Mean fluorescence intensity (MFI) analysis shows reduced protein glycosylation in hepatocytes from N-LKO mice compared to WT mice fed a chow diet (n = 4), as indicated by staining with Wheat Germ Agglutinin (WGA), a lectin that specifically binds to glycosylated carbohydrate residues on proteins. **(C)** NgBR expression was suppressed by transfecting Huh7 cells with specific siRNA against NgBR. **(D)** The histogram and MFI illustrate the reduction in protein glycosylation in Huh7 cells upon knocking down NgBR (ShNgBR) compared to the control (ShC), as revealed by staining with WGA, a lectin that binds to carbohydrate. **(E)** Images of primary hepatocytes isolated from N-LKO mice fed a chow diet show improved survival after 24-hour culture in serum-free medium containing 0.2% BSA and dolichol-P (50 ug/mL). In contrast, cells cultured in serum-free medium with 0.2% BSA without dolichol-P show no survival, while those cultured in 10% FBS serve as a positive control for cell survival. Two-sided $^{*}P < 0.05$; comparing N-LKO with WT mice using an unpaired Welch's t-test. $^{*}P < 0.05$, Huh7-shNgBR vs. Huh7-shC; $^{***}P < 0.001$, Huh7-shNgBR vs. Huh7-shC.

Fig. S14

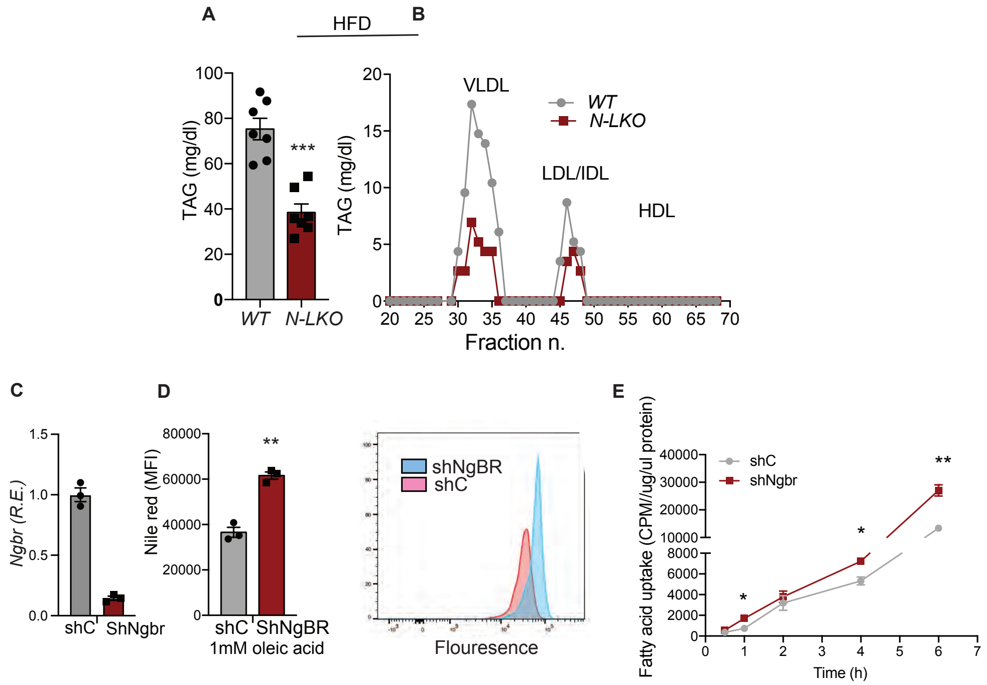


**Figure S14. Loss of NgBR function in hepatocytes leads to reduced circulating triglycerides and enhanced lipid uptake by hepatocytes. (A)** Circulating TAG levels were reduced in overnight fasted N-LKO mice compared to WT mice after 4 months of fed high-fat-diet (HFD) feeding (n=7). **(B)** TAG content of FPLC-fractionated lipoproteins from pooled plasma (n=5) of overnight-fasted N-LKO mice was reduced compared to WT mice (n=5) after 4 months of HFD feeding. **(C)** Stable transfection of Huh7 cells with shRNA targeting NgBR: qRT-PCR results depicting reduced NgBR expression upon knockdown. R.E., relative expression. **(D)** shNgBR-Huh7 and control shC-Huh7 cells were treated with 1mM oleic acid for 24 hours. Neutral lipid accumulation, as indicated by Nile red staining, and mean fluorescence intensity (MFI) determined by flow cytometry. **(E)** Radiolabeled Oleic acid uptake over time in Huh7 cells, showing increased fatty acid absorption after stable knockdown of NgBR. Statistical significance: **P < 0.01; ***P < 0.001, using Welch's t-test and *P< 0.05; **P < 0.01, and ***P < 0.001 using two-way ANOVA with Sidak's multiple comparisons test.

Fig. S15

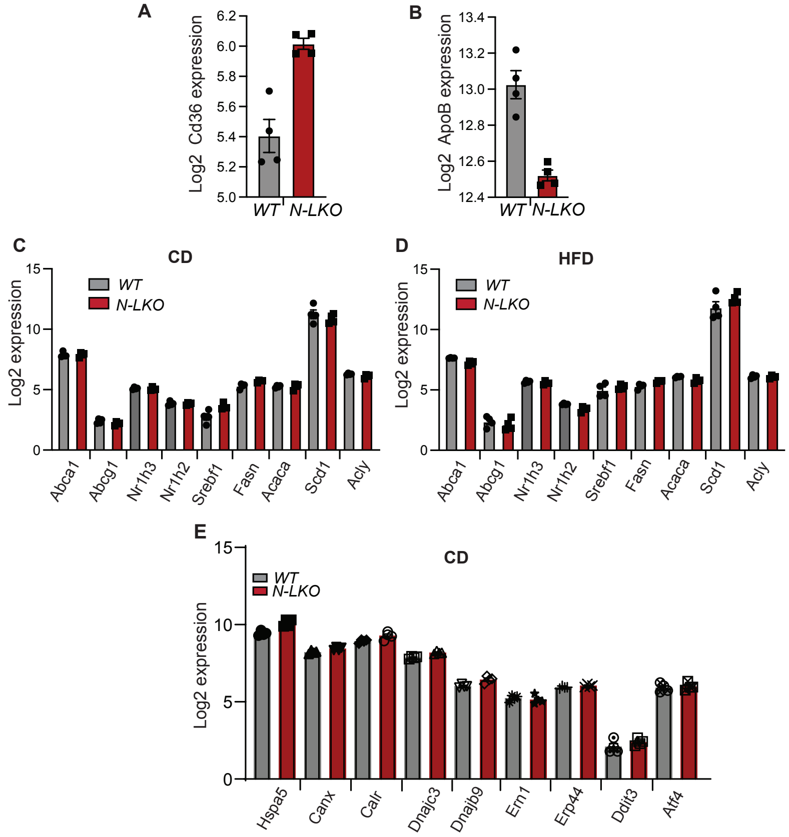


**Figure S15. Hepatocyte-specific NgBR deletion increases lipid uptake gene expression and decreases VLDL secretion gene expression, without affecting LXR pathway or basal ER stress genes. (A)** Log-transformed mRNA levels of Cd36 in the livers of WT and N-LKO mice fed a chow diet for 8 weeks. **(B)** Log-transformed mRNA expression of ApoB in the livers of WT and N-LKO mice on an HFD for 16 weeks. **(C-D)** Log-transformed mRNA levels of LXR target genes in the livers of WT and N-LKO mice. Mice were fed either a chow diet (CD) for 8 weeks **(C)** or a high-fat diet (HFD) for 16 weeks **(D)**. (n=4)**. (E)** Log-transformed mRNA levels of ER stress-related genes in livers of WT and N-LKO mice fed a chow diet for 8 weeks. (n = 4 mice per group)
