# Supplementary figures and images for: Impaired glycosylation promotes rapid transition to hepatocellular carcinoma in model of diet-induced steatotic liver disease
Source: J Clin Invest. 2026 Mar 10;136(9):e197719. doi: 10.1172/JCI197719 (PMC13132402; doi:10.1172/JCI197719)

Fig.4D

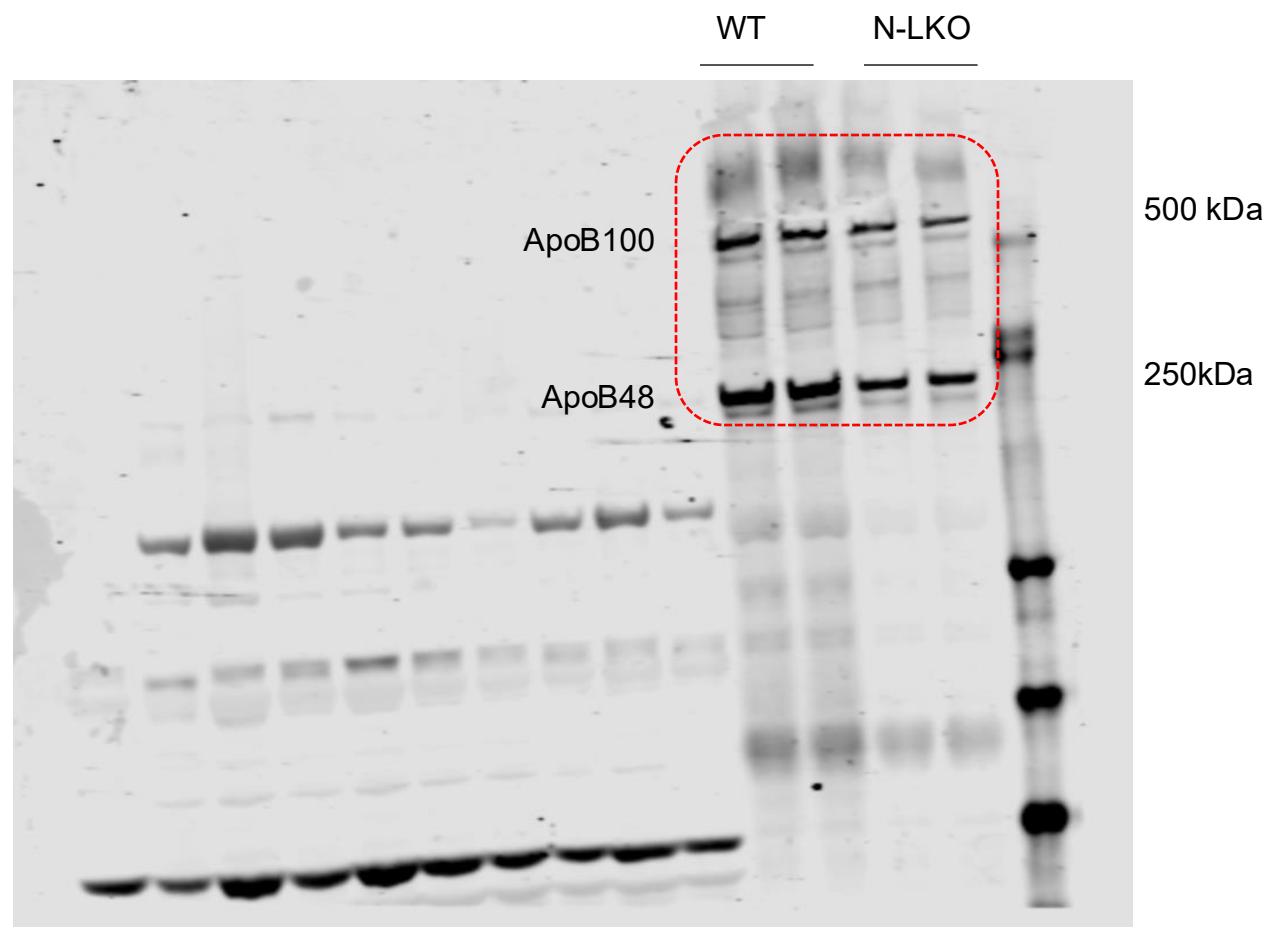

Fig. 5D

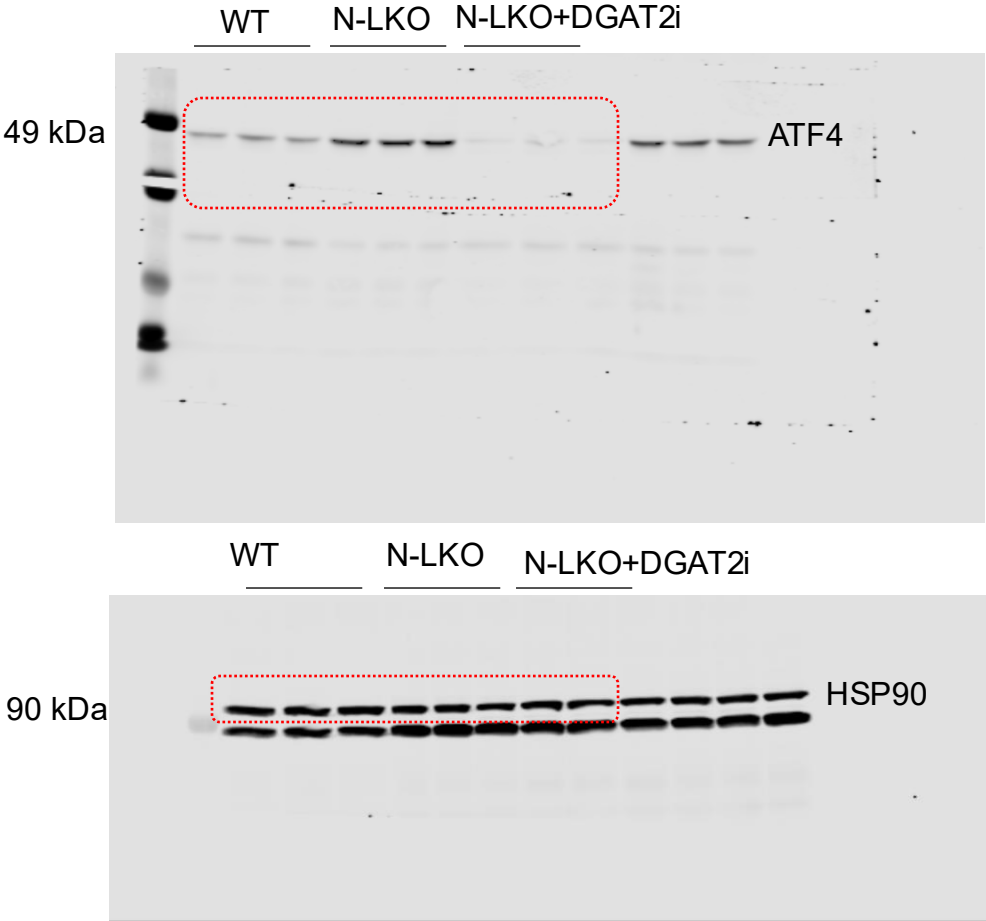

Fig. S12C

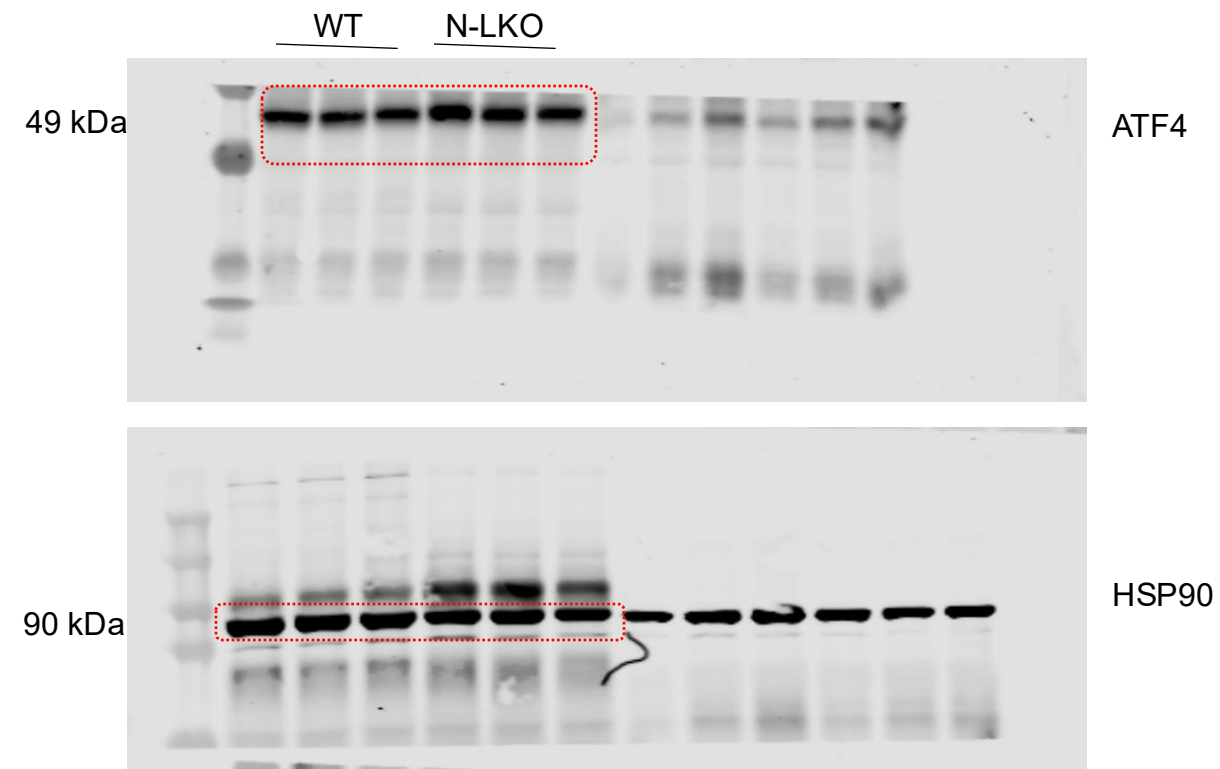

Fig. S1A

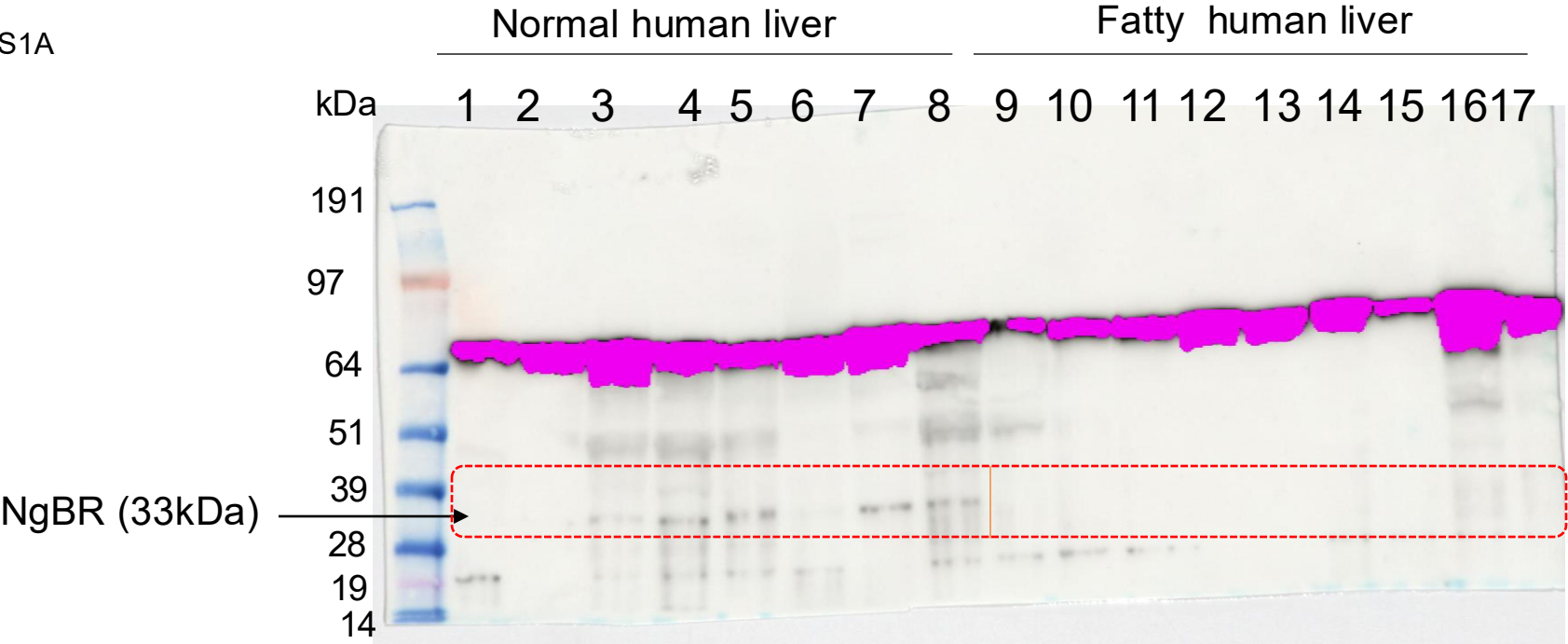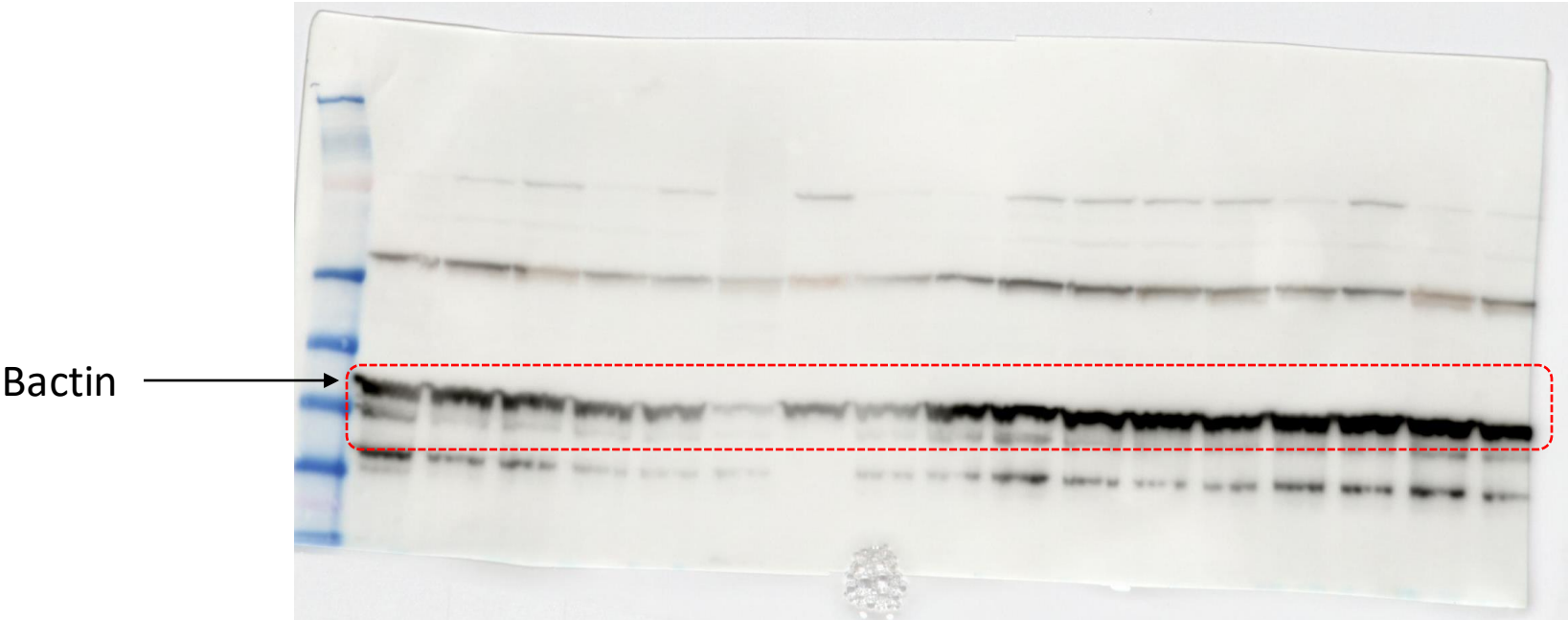

Supplement: Unedited blot and gel images [file jci-136-197719-s336.pdf]
